# Supplementary material for: In situ-formed tetrahedrally coordinated double-helical metal complexes for improved coordination-activated n-doping
Source: Nat Commun. 2022 Mar 8;13:1215. doi: 10.1038/s41467-022-28921-5 (PMC8904628; doi:10.1038/s41467-022-28921-5)
Supplement: Supplementary file 1 — Supplementary Information [file 41467_2022_28921_MOESM1_ESM.pdf]

Supplementary Information

In situ-formed tetrahedrally coordinated double-helical metal complexes for improved coordination-activated n-doping

Ziyang Liu[1], Xiao Li[1], Yang Lu[2], Chen Zhang[1], Yuewei Zhang[1,3], Tianyu Huang[1], Dongdong Zhang[1,3] * & Lian Duan[1,3] *

[1] Key Lab of Organic Optoelectronics and Molecular Engineering of Ministry of Education, Department of Chemistry, Tsinghua University, Beijing 100084, China.

[2] Institute of Drug Discovery Technology, Ningbo University, Ningbo 315211, China.

[3] Center for Flexible Electronics Technology, Tsinghua University, Beijing 100084, China.

*E-mail: ddzhang@mail.tsinghua.edu.cn; duanl@mail.tsinghua.edu.cn

Table of Contents

1. Supplementary Figures 1–30

2. Supplementary Tables 1–7

3. Supplementary Methods

3.1 General information

3.2 Thermal properties measurement

3.3 Electrochemical Measurement

3.4 Synthesis of diphenanthroline derivatives

4. Supplementary References

1. Supplementary Figures 1–30

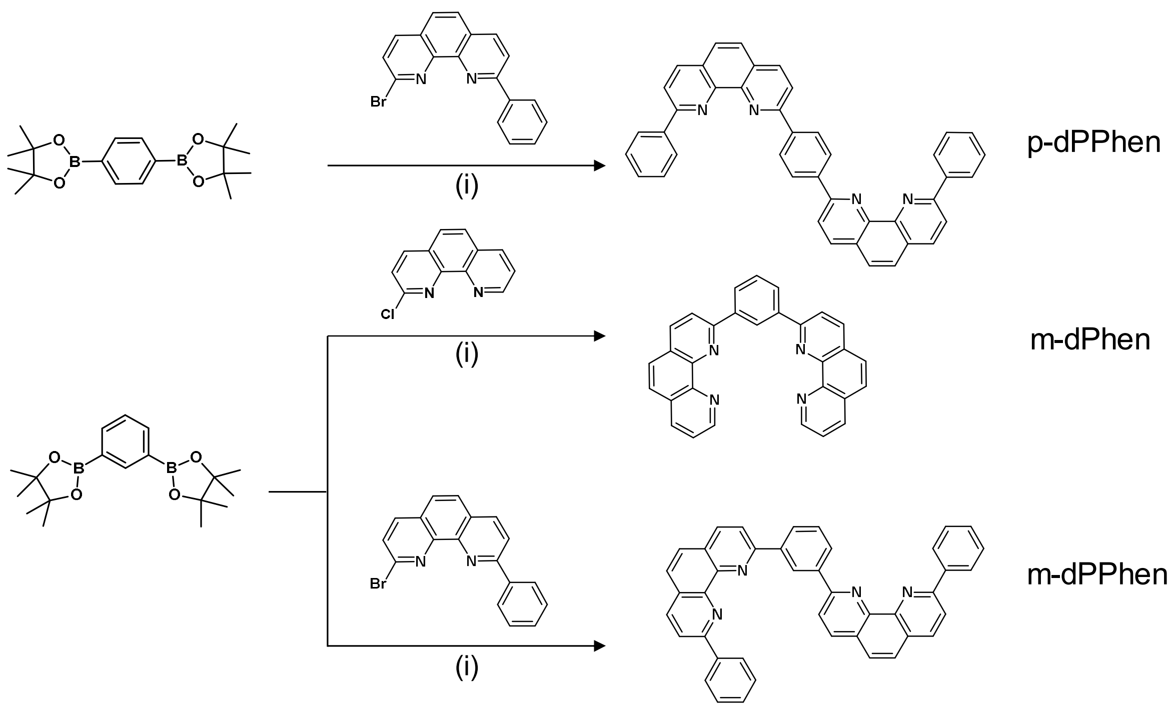

(i) $Pd(PPh_3)_4$, Na_2CO_3, Toluene/EtOH/H_2O, reflux, 100 °C, 30 h

Supplementary Figure 1. The synthesis routes of diphenanthroline derivatives.

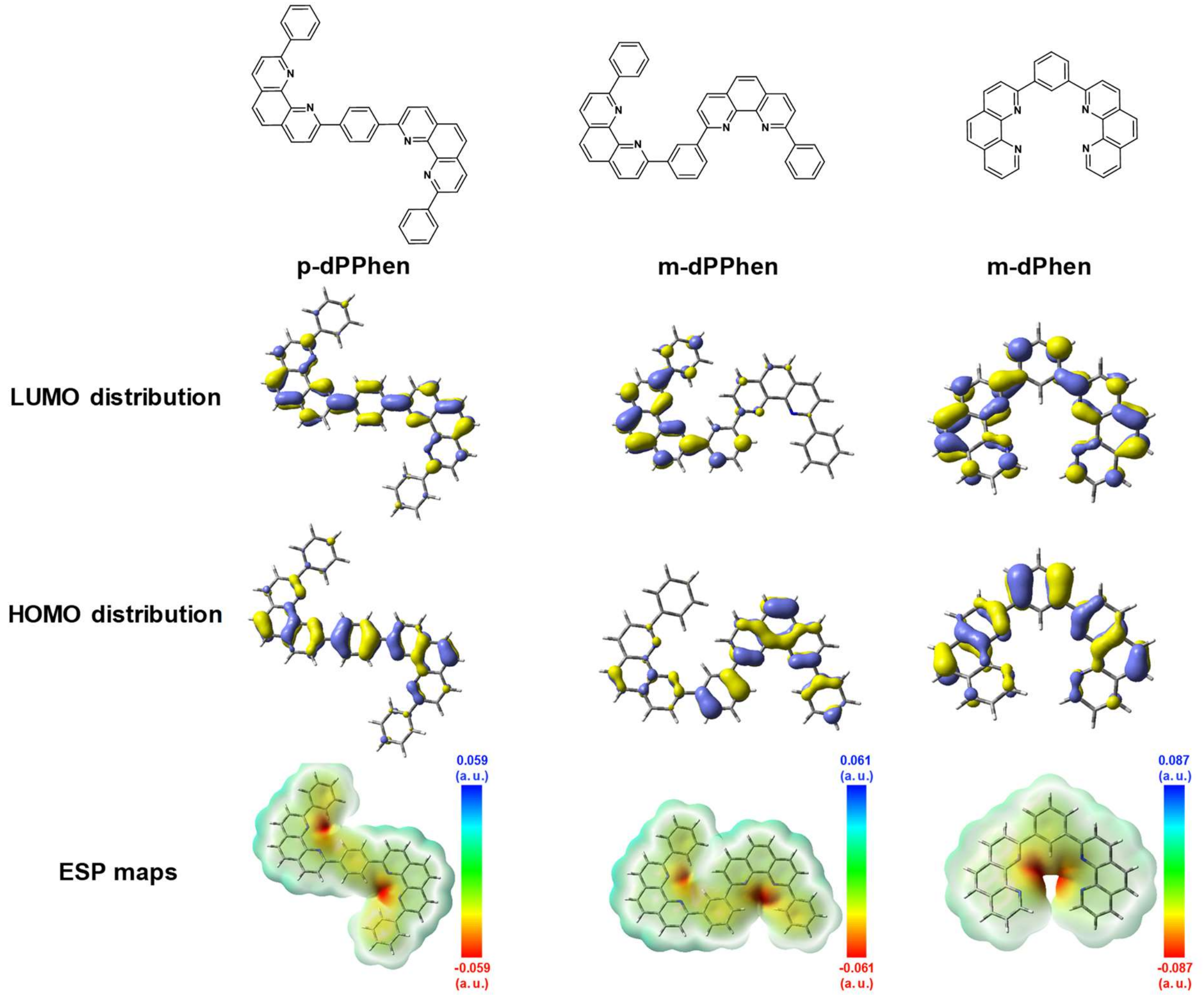

Supplementary Figure 2. The optimized geometries, HOMO/LUMO distributions and electrostatic potential surfaces of three diphenanthroline derivatives.

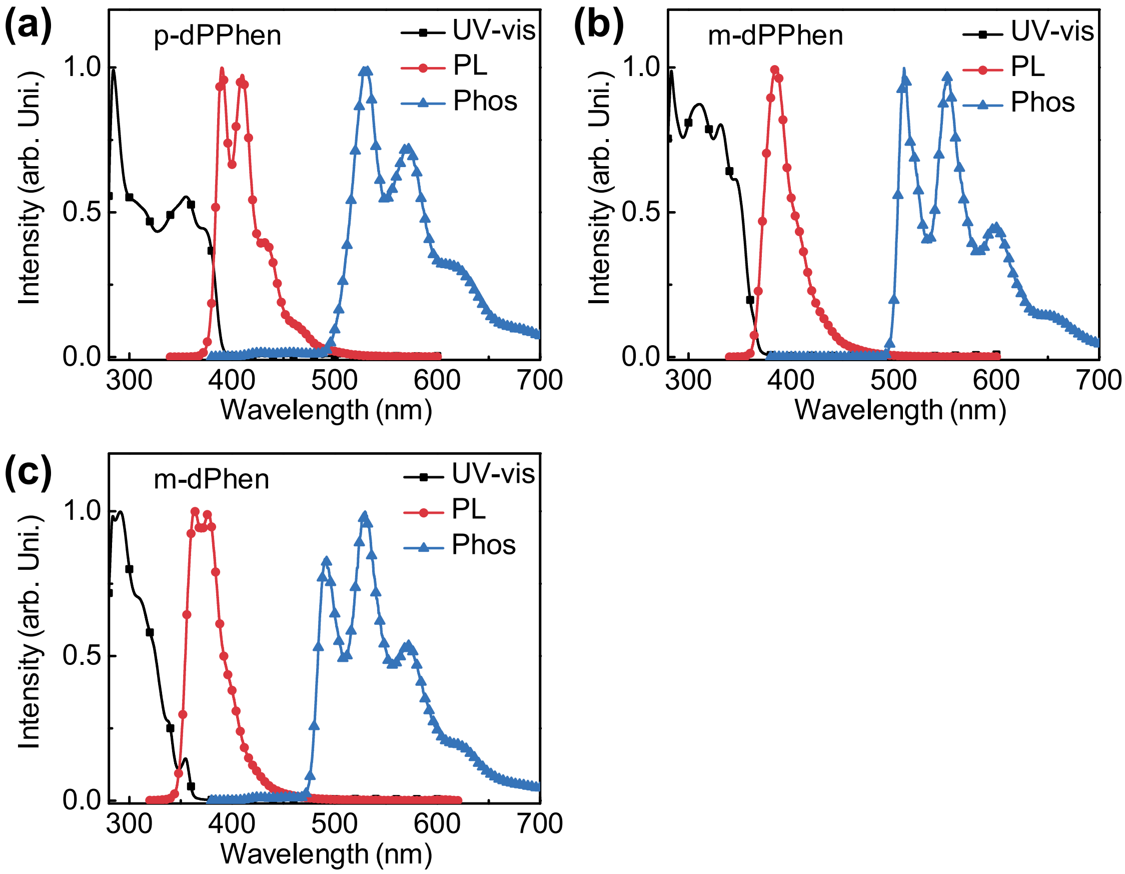

Supplementary Figure 3. The absorption, fluorescence and phosphorescence spectra of three diphenanthroline derivatives in toluene solution with a concentration about 10^{-5} mol L^{-1}.

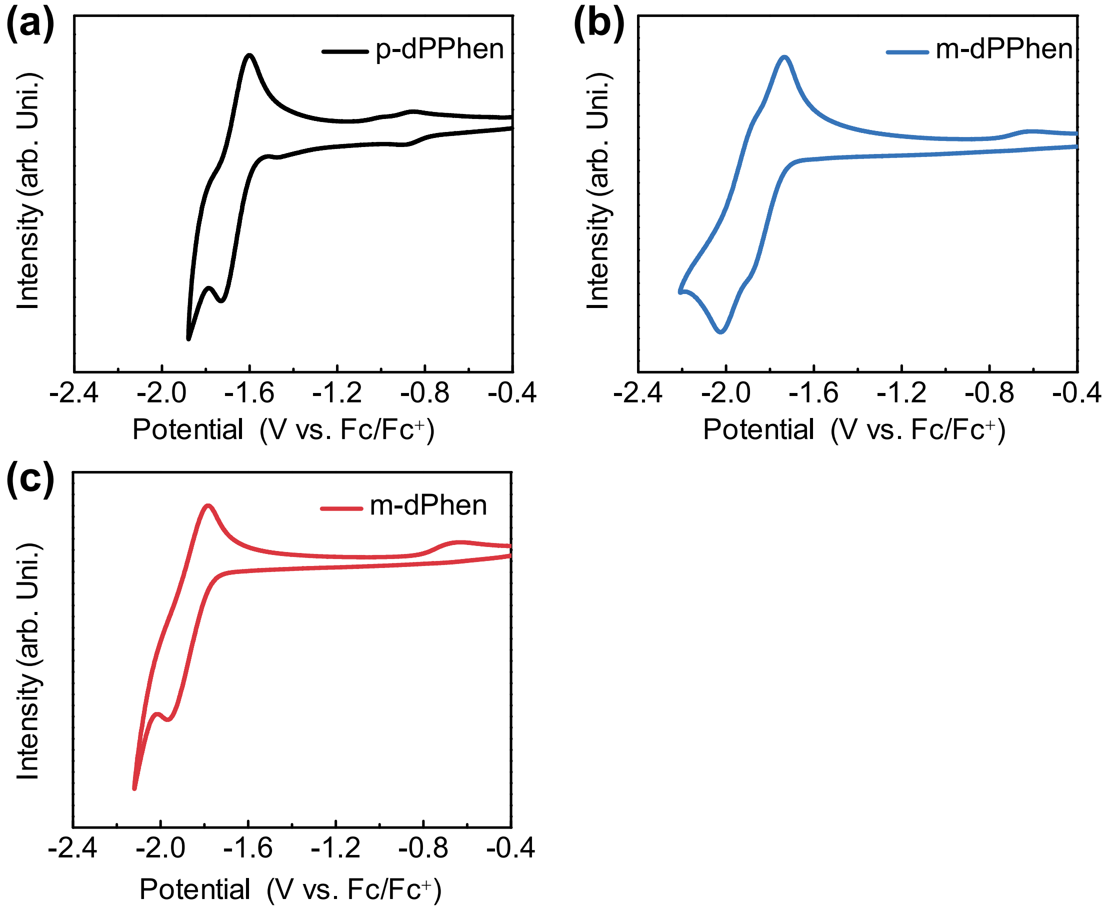

Supplementary Figure 4. The Cyclic voltammetry (CV) curves of three diphenanthroline derivatives. (a) The CV curves of p-dPPhen. (b) The CV curves of m-dPPhen. (c) The CV curves of m-dPhen. Different from p-dPPhen and m-dPhen with only one redox, the m-dPPhen shows two redox couples. This can be understood because the adjacent 2PPhen groups in m-dPPhen have moderate difference in electron acceptor properties and lead to two redox couples within the range of applied bias. The chemical environments of two 2PPhen groups for p-dPPhen and two Phen groups for m-dPhen are almost identical due to the symmetrical configurations, thus showing one main redox in CV measuremnt.

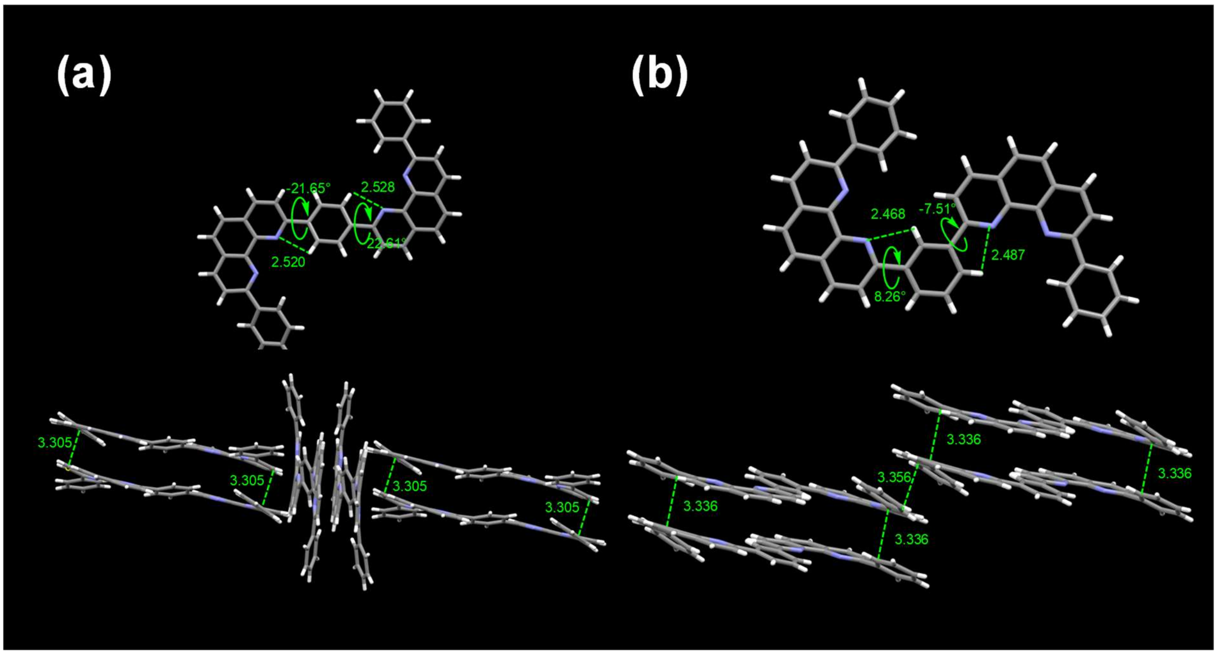

Supplementary Figure 5. The single crystal structures and packing models of (a) p-dPPhen and (b) m-dPPhen. The CCDC reference numbers of p-dPPhen (left) and m-dPPhen (right) are 2088633 and 2088642, respectively.

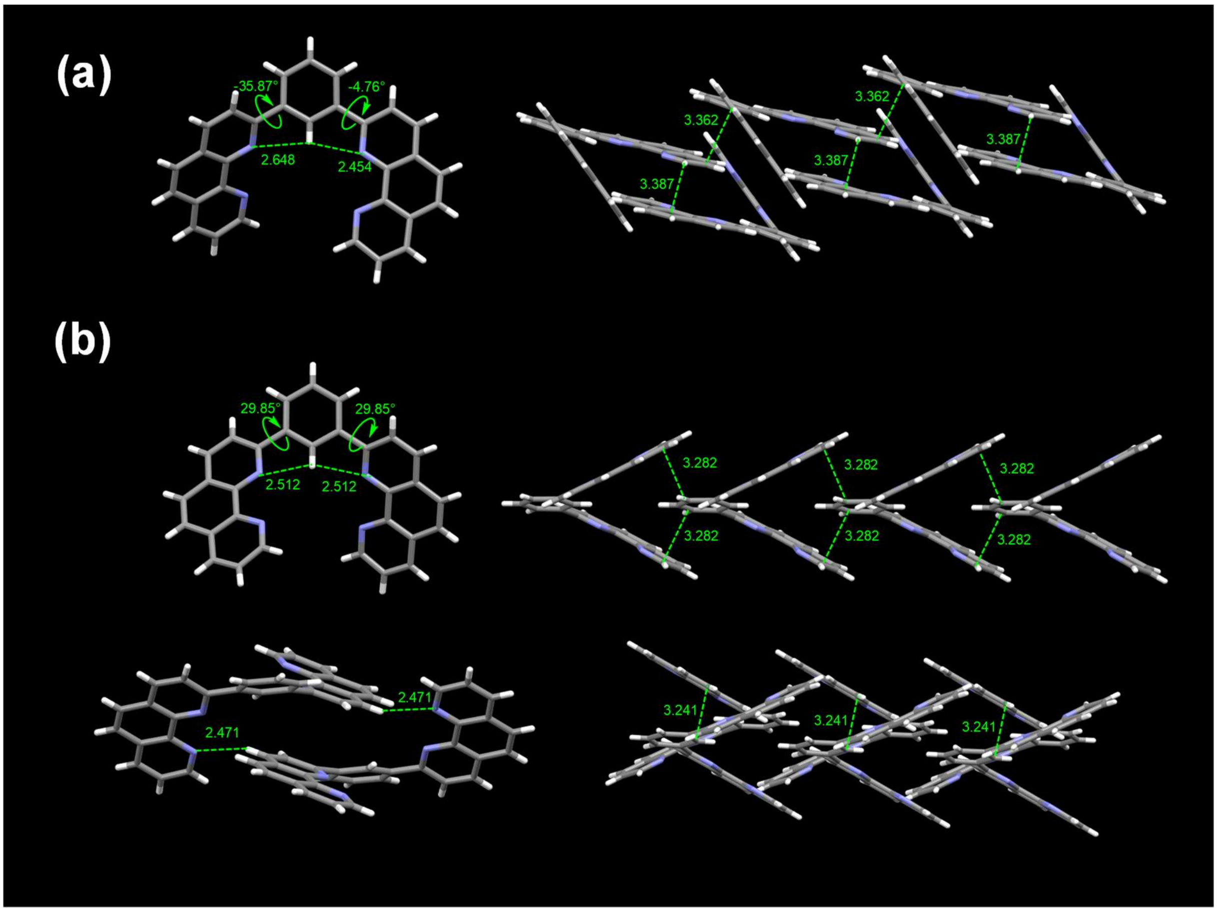

Supplementary Figure 6. The single crystal structures and packing models of m-dPhen. The single crystals of m-dPhen are obtained by (a) slow evaporation of CH_2Cl_2/EtOAc solution and (b) train sublimation. The CCDC reference numbers of m-dPhen obtained by solvent evaporation and sublimation are 2088638 and 2088639, respectively.

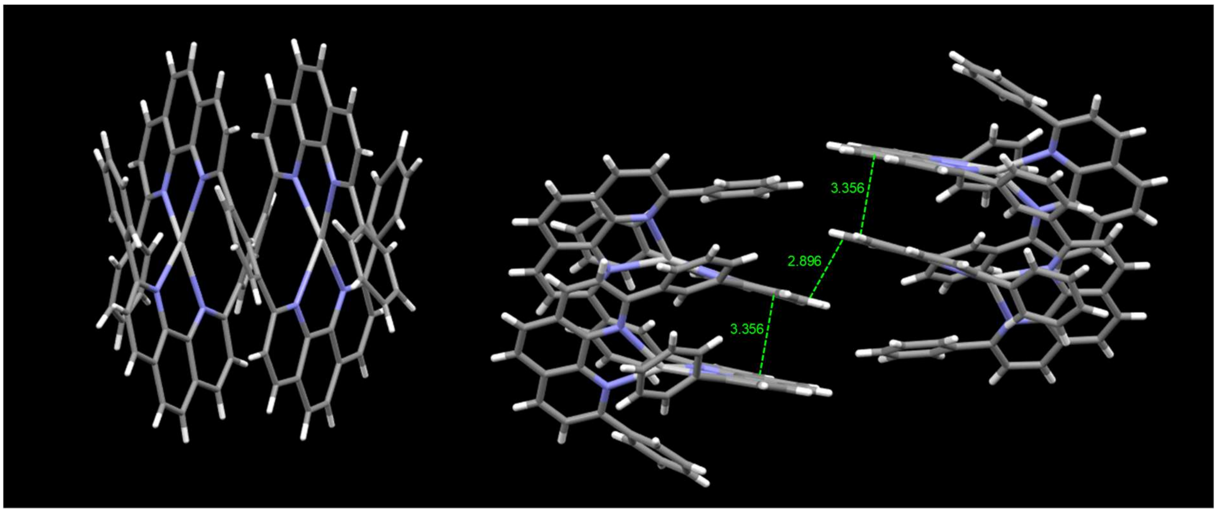

Supplementary Figure 7. The single crystal structures and packing models of $[Ag_2(m\text{-}dPPhen)_2](NO_3)_2$. The anions of NO_3^- are omitted to better present the crystal structures. The CCDC reference numbers of $[Ag_2(m\text{-}dPPhen)_2](NO_3)_2$ is 2088643.

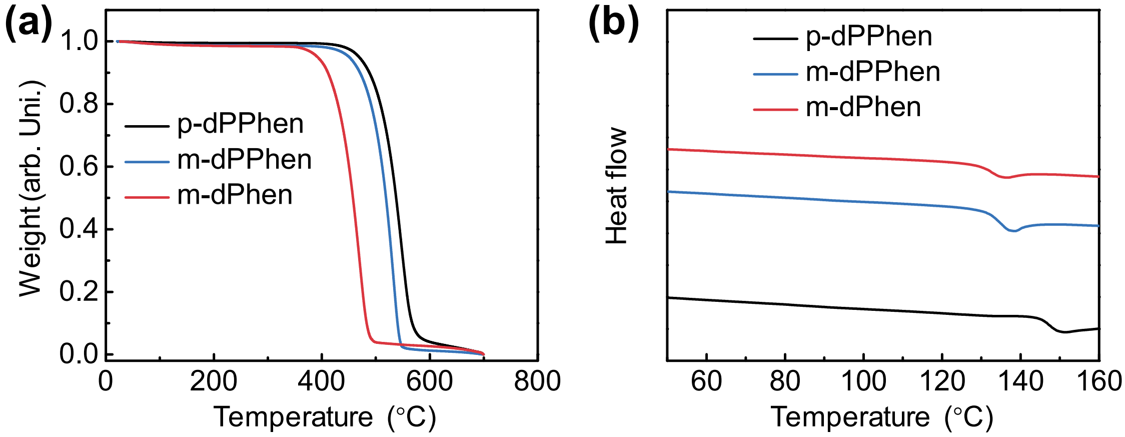

Supplementary Figure 8. The (a) TGA and (b) DSC curves of four diphenanthroline derivatives with a temperature rising rate of 10 °C min^{-1}.

(a)

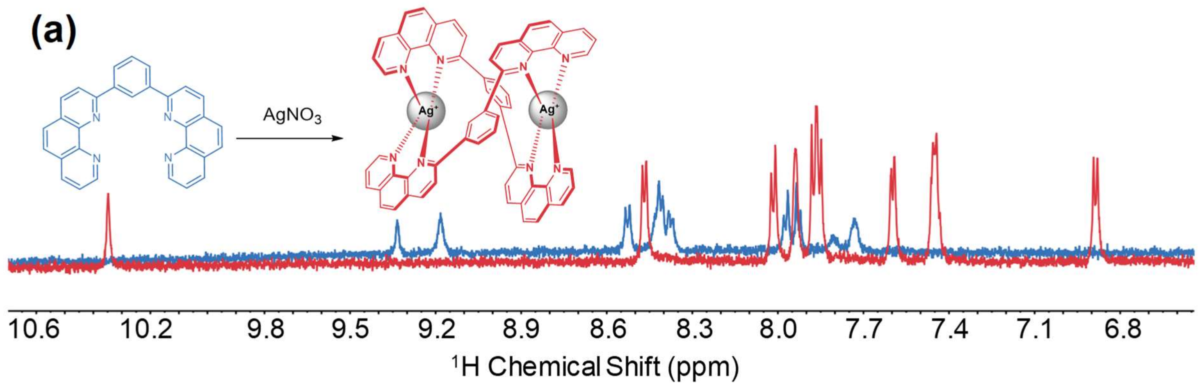

(b)

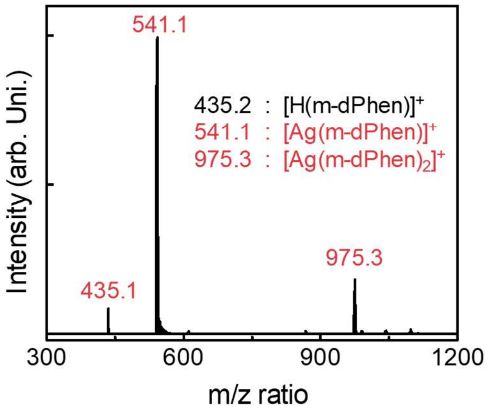

Supplementary Figure 9. (a) ^{1}H NMR spectra of m-dPhen (blue line) and resulting products (red line) of m-dPhen:$AgNO_3$ (molar ratio of 1:1) in CD_3CN. The ^{1}H NMR measurement have been successfully applied to interaction between organic base and ETMs in OLEDs[1,2]. Herein, the ^{1}H-NMR of pristine m-dPhen shows significant downward shift after mixed with $AgNO_3$. The similar phenomena have also been observed for the coordination reactions between multi-1,10-phenanthroline ligands with 1,3-phenylene linkers and metals including lithium, copper and silver[3,4], which indicated the formation of tetrahedrally coordinated double-helical metal complex between m-dPhen and $AgNO_3$. (b) The MALDI-TOF mass spectrum of m-dPhen:Ag (130 nm, 10 wt%) films. Given that the relative molecular mass of m-dPhen ($C_{30}H_{18}N_4$) and relative atomic mass of silver atom (Ag) are 434.50 and 107.87, the molar ratio of m-dPhen:Ag in doped films (10 wt%, weight ratio of m-dPhen:Ag = 10:1) is calculated to be 2.48:1. Due to the strong coordination ability of m-dPhen with favorable molecular configuration and reduced steric hinderance, the species in m-dPhen:Ag (130 nm, 10 wt%) films are expected to be dominated by coordination products of $[Ag(m\text{-}dPhen)]^+$ and $[Ag(m\text{-}dPhen)_2]^+$, which is in accordance with the results of Supplementary Fig. 9b. Additionally, for p-dPPhen ($C_{42}H_{26}N_4$) and m-dPPhen ($C_{42}H_{26}N_4$) with molecular weight of 586.70, the molar ratio of p-dPPhen:Ag (130 nm, 10 wt%) and m-dPPhen:Ag (130 nm, 10 wt%) films is calculated to be 1.84:1. It can be founded that although the Ag-doping concentration (molar ratio) of p-dPPhen, m-dPPhen and m-dPhen is almost identical, the m/z peak of prisitine p-dPPhen in doped films is obviously higher than those of pristine m-dPPhen and m-dPhen (Fig. 2d and Supplementary Fig. 9b), verifying the influence of π-linkages in diphenanthroline ligands on their different coordination abilities with silver.

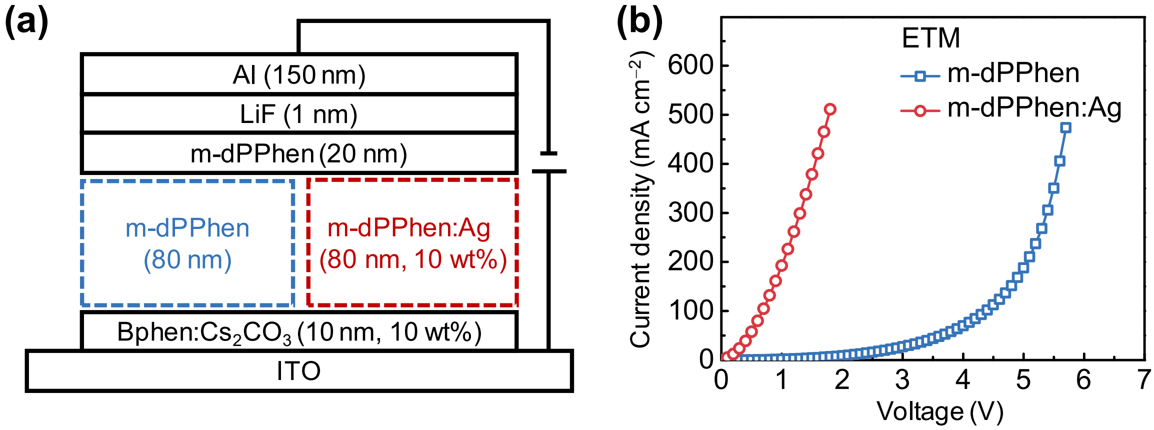

Supplementary Figure 10. (a) The device structure of EODs with undoped/Ag-doped m-dPPhen (80 nm) as ETMs. (b) The J–V characteristics of structures of EODs with undoped/Ag-doped m-dPPhen (80 nm) as ETMs. With the same electron injection layer of m-dPPhen (20 nm)/LiF (1 nm)/Al (150 nm), the different J–V characteristics of abovementioned two EODs should be attributed to the electron transport abilities of pristine m-dPPhen (80 nm) and m-dPPhen:Ag (80 nm, 10 wt%). As plotted in Supplementary Fig. 10, the current density of EODs based on ETMs of m-dPPhen:Ag is significantly higher than that of EODs based on pristine m-dPPhen, which indicates the superior electron transport ability of m-dPPhen:Ag (10 wt%). Therefore, the Ag-doped diphenanthroline can be adopted as both efficient EILs and ETMs.

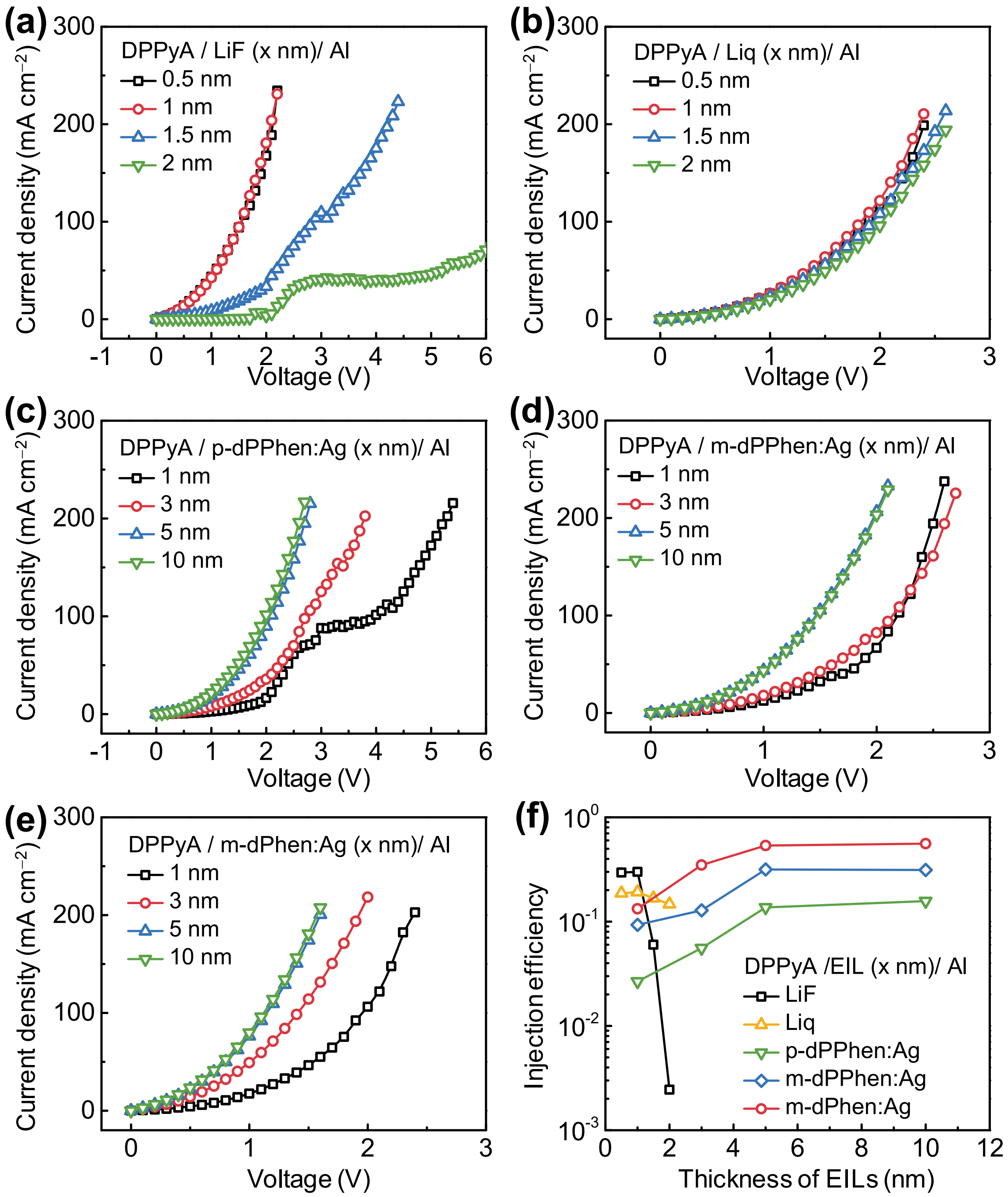

Supplementary Figure 11. (a) The J–V characteristics of EODs with LiF at thicknesses of 0.5, 1, 1.5 and 2 nm. (b) The J–V characteristics of EODs with Liq at thicknesses of 0.5, 1, 1.5 and 2 nm. (c) The J–V characteristics of EODs with p-dPPhen:Ag at thicknesses of 1, 3, 5 and 10 nm. (d) The J–V characteristics of EODs with m-dPPhen:Ag at thicknesses of 1, 3, 5 and 10 nm. (e) The J–V characteristics of EODs with m-dPhen:Ag at thicknesses of 1, 3, 5 and 10 nm. (f) The Injection efficiency-thickness of different EILs. The optimal thickness of typical LiF and Liq was set as 1 nm so that thermally hot Al atom can react with isolated clusters of lithium compound and ETMs. Because the electron injection enhancement of Ag-doped EILs was attributed to the work function downshifts of cathode modification, the formation homogeneous surface with high coverage is essential to enable efficient cathode modification. Based on these, lithium compounds with thickness of 1 nm and Ag-doped diphenanthroline ligands of 5 nm were employed in Figs. 3c and 3d to compare the injection properties.

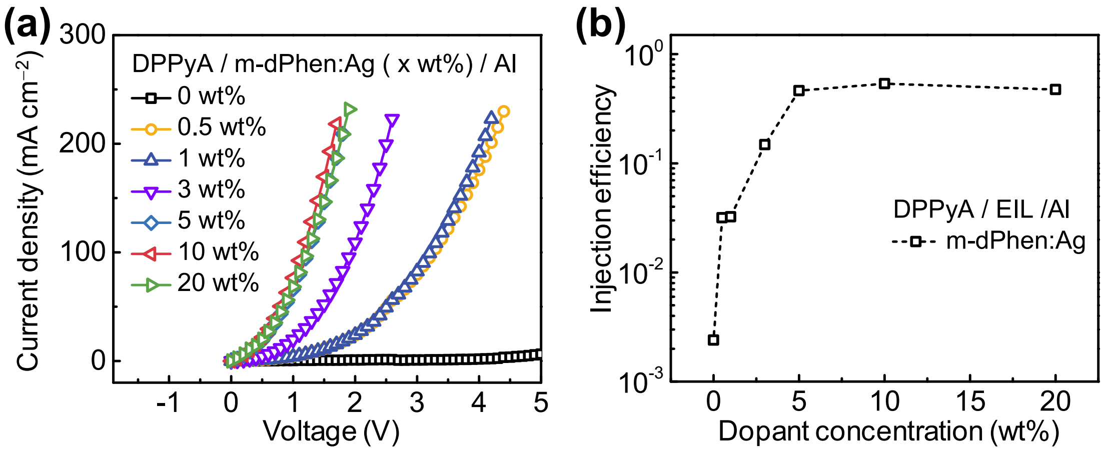

Supplementary Figure 12. (a) The *J*–*V* characteristics of EODs with structures of ITO/Bphen:Cs_2CO_3 (10 nm, 10 wt%)/ DPPyA (100 nm)/m-dPhen:Ag (0, 0.5, 1, 3, 5, 10 and 20 wt%, 5 nm)/Al (150 nm). (b) Injection efficiency of EODs based on different EILs of m-dPhen:Ag (0, 0.5, 1, 3, 5, 10 and 20 wt%, 5 nm). The electron injection efficiency of m-dPhen:Ag at lower concentration of 5 wt% (0.5 vol%) is very close to those at 10 wt% (1 vol%) and 20 wt% (2 vol%), which can be interpreted by the strong coordination ability of m-dPhen.

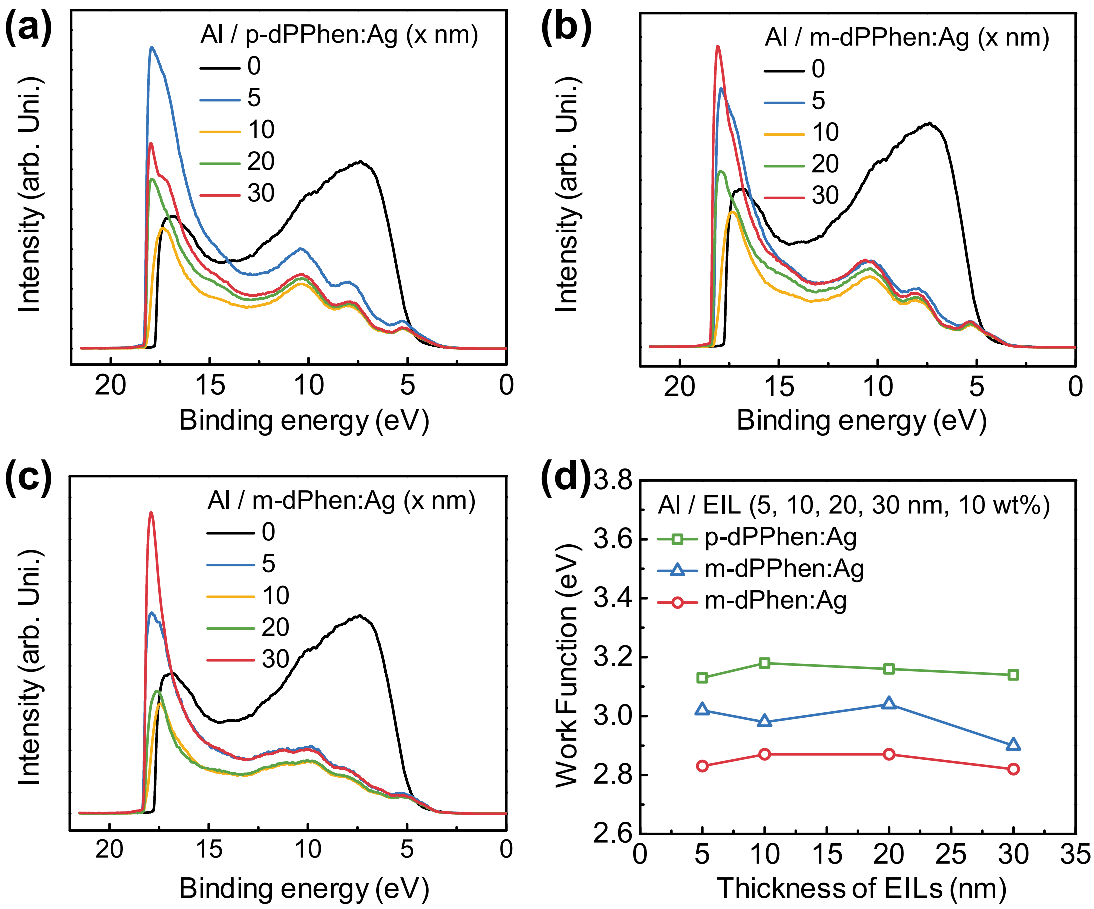

Supplementary Figure 13. (a) UPS data of aluminum cathode modified by p-dPPhen:Ag with thickness of 0, 5, 10, 20 and 30 nm. (b) UPS data of aluminum cathode modified by m-dPPhen:Ag with thickness of 0, 5, 10, 20 and 30 nm. (c) UPS data of aluminum cathode modified by m-dPhen:Ag with thickness of 0, 5, 10, 20 and 30 nm. (c) The work functions of aluminum cathode modified by different Ag-doped EILs with thickness of 5, 10, 20 and 30 nm.

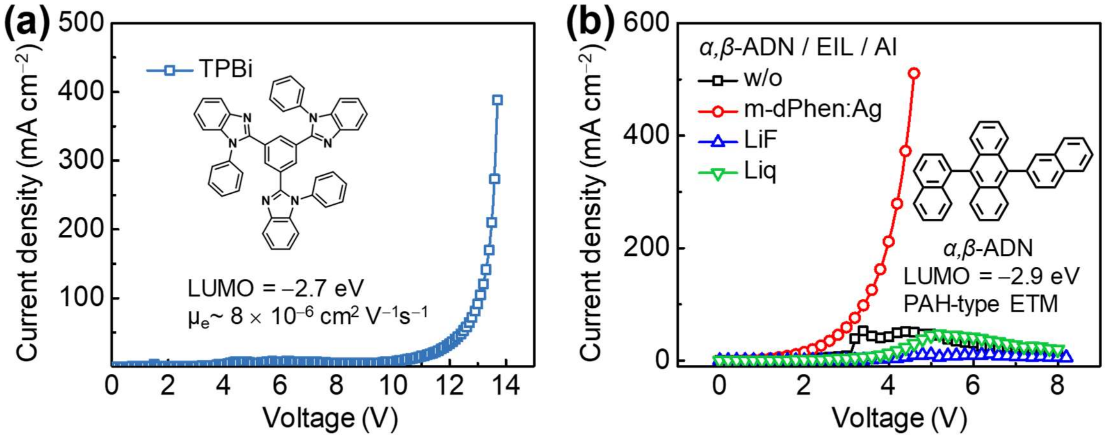

Supplementary Figure 14. (a) The J–V characteristics of EODs with structures of ITO/Bphen:Cs_2CO_3 (10 nm, 10 wt%) /TPBi (100 nm) /m-dPhen:Ag (5 nm, 10 wt%)/Al (150nm). (b) The J–V characteristics of EODs with structures of ITO/Bphen:Cs_2CO_3 (10 nm, 10 wt%)/*α,β*-ADN (100 nm)/EIL of w/o, LiF (1 nm), Liq (1 nm) and m-dPhen:Ag (5 nm, 10 wt%)/Al (150 nm). Accoriding to previous reports[1,5], the coordination reaction at the interface of nitrogen-containing ETM/EIL of lithium compound (1 nm)/Al is essential for the work function tuning and good electron injection properties of LiF or Liq. As a result, the electron injection property of LiF/Al is rather poor when replacing typical nitrogen-containing ETMs with nitrogen-free polycyclic aromatic hydrocarbon (PAH) compounds such as *α,β*-ADN and ADN with deep LUMO levels. For the same nitrogen-free ETM of *α,β*-ADN, the electron injection property of m-dPhen:Ag (5 nm, 10 wt%) is much better than the lithum compounds including LiF and Liq (Supplementary Fig. 12b), which can be understood by the small energy offset between the LUMO level (–2.9 eV) of *α,β*-ADN and work function (2.8 eV) of Al/m-dPhen:Ag (5 nm, 10 wt%). For the nitrogen-containing ETMs of DPPyA, the electron injection property of LiF is slightly lower than the m-dPhen:Ag (5 nm, 10 wt%) due to the existance of coordination reaction between DPPyA/LiF/Al (Fig. 3c and 3d). Therefore, the J–V characteristics of EODs based on ETMs of *α,β*-ADN confirms that CAN strategies enable efficient electron injection for various ETMs.

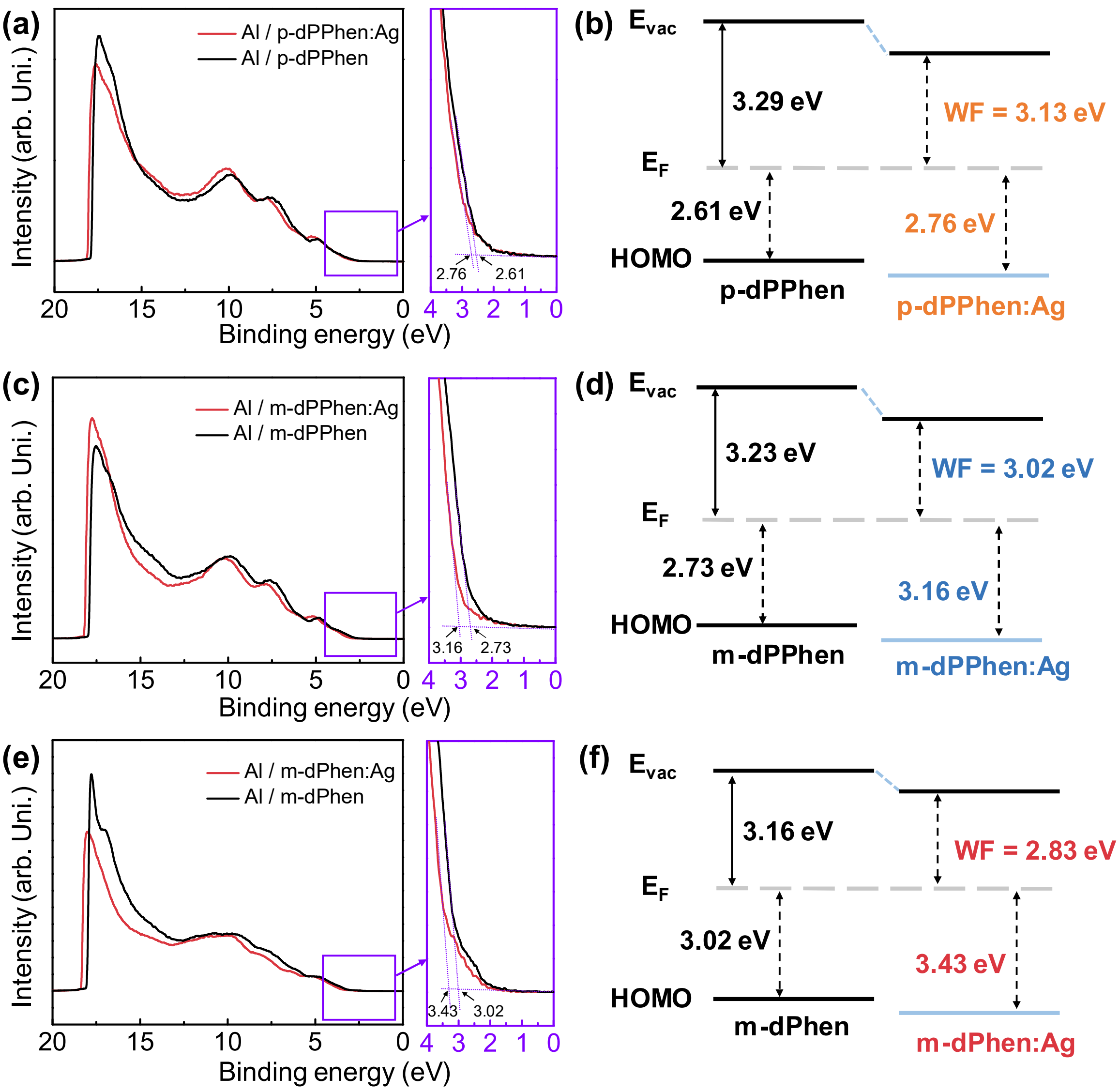

Supplementary Figure 15. Ultraviolet photoelectron spectroscopy (UPS) analysis and schematic energy-level diagrams of Al cathodes modified by 5 nm of prinstine or Ag-doped diphenanthroline films. (a) UPS analysis and (b) schematic energy-level diagrams of Al (10 nm)/p-dPPhen (5 nm) and Al (10 nm)/p-dPPhen:Ag (5 nm, 10 wt%). (c) UPS analysis and (d) schematic energy-level diagrams of Al (10 nm)/m-dPPhen (5 nm) and Al (10 nm)/m-dPPhen:Ag (5 nm, 10 wt%). (e) UPS analysis and (f) Schematic energy-level diagrams of Al (10 nm)/m-dPhen (5 nm) and Al (10 nm)/m-dPhen:Ag (5 nm, 10 wt%). The HOMO positions were determiend by the intersection of the two tangent lines (in purple) at the low binding energy region. According to the results of UPS analysis, the energy offsets between E_F and HOMO level increased after Ag-doping, which verifying the coordination-activated n-doping effect.

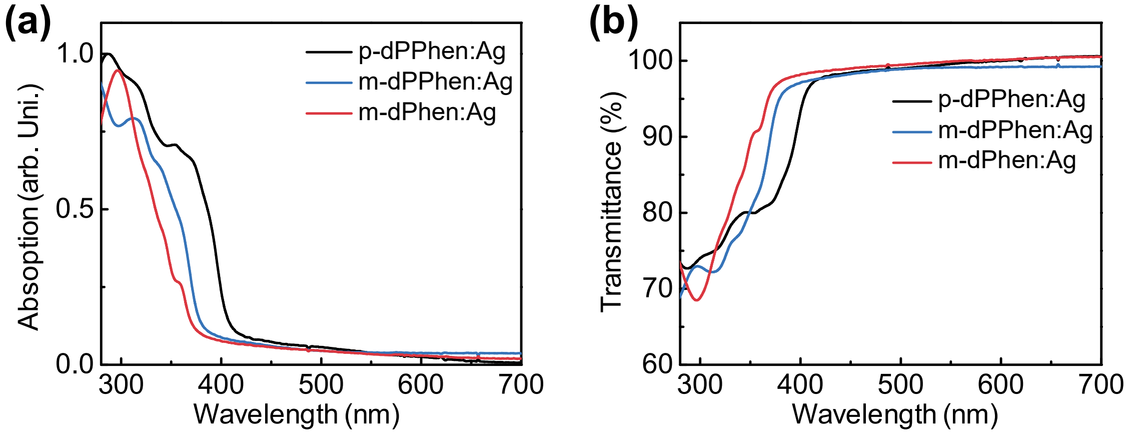

Supplementary Figure 16. (a) The normalized absorption spectra of p-dPPhen:Ag (10 wt%, 10 nm), m-dPPhen:Ag (10 wt%, 10 nm) and m-dPhen:Ag (10 wt%, 10 nm), respectively. (b) The light transmittances of the p-dPPhen:Ag (10 wt%, 10 nm), m-dPPhen:Ag (10 wt%, 10 nm) and m-dPhen:Ag (10 wt%, 10 nm), respectively.

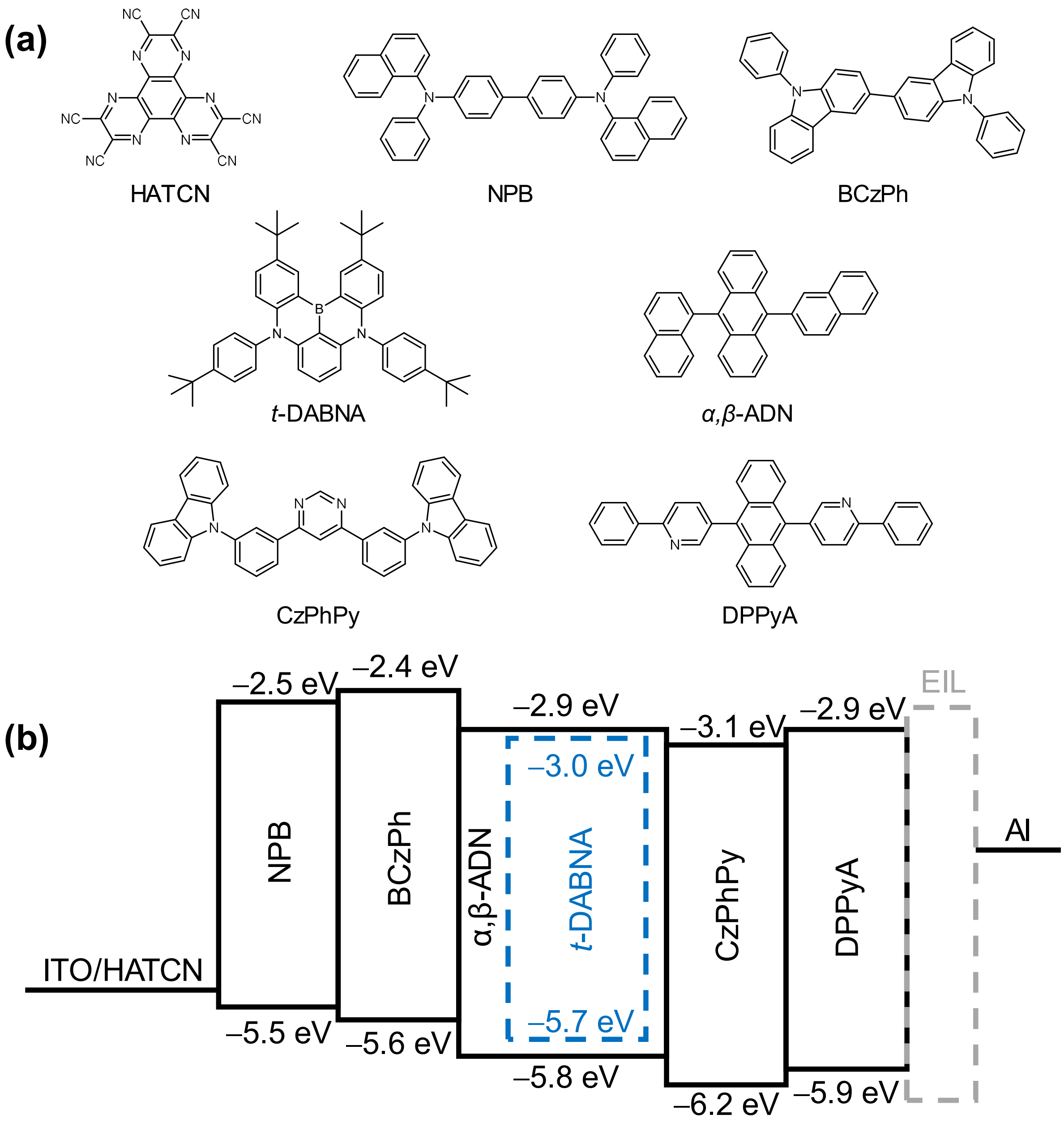

Supplementary Figure 17. (a) The chemical structures of materials used in bottom-emitting deep-blue OLEDs. (b) The energy levels of the deep-blue OLEDs

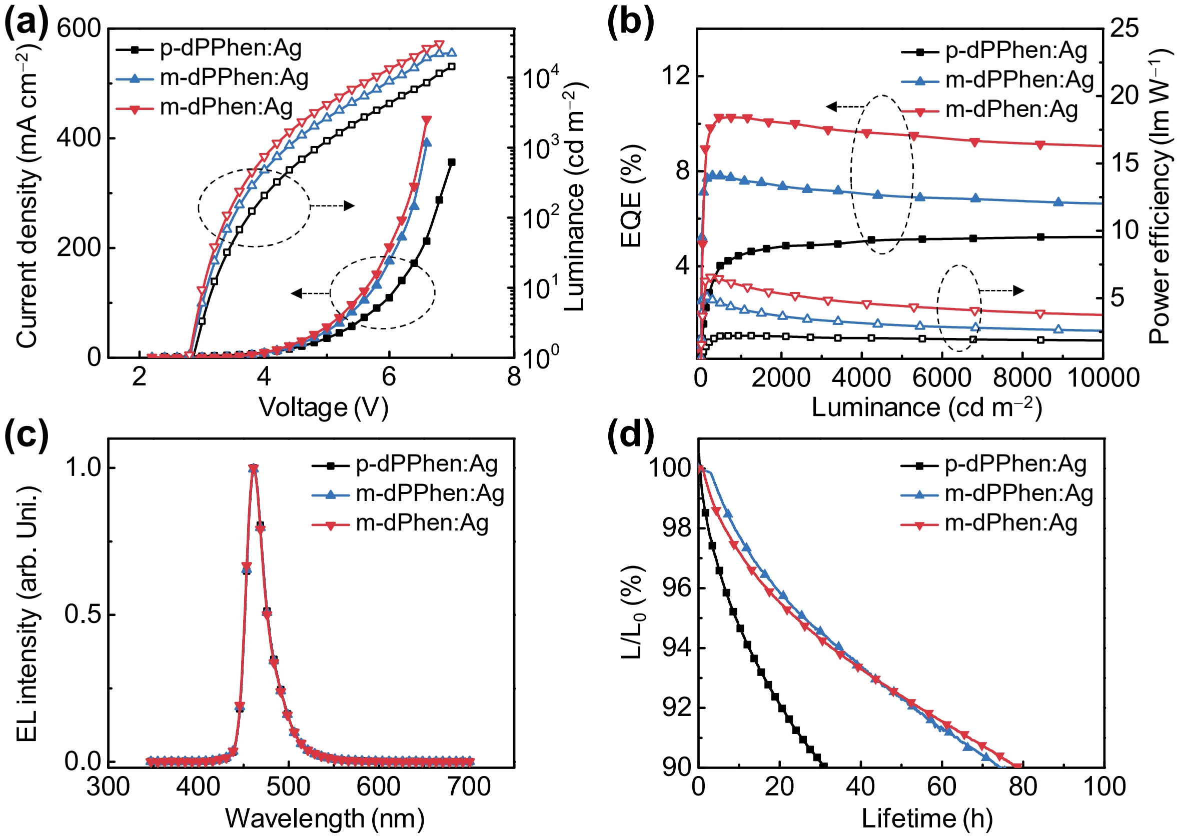

Supplementary Figure 18. The performances of OLEDs using different Ag-doped diphenanthroline ligands as EIL. (a) The current density–luminance–voltage of OLED devices using different EILs. (b) The EQE–power efficiency–luminance of OLED devices using different EILs. (c) The EL spectra at 1000 cd m^{-2}. (d) The lifetimes measured under 2000 cd m^{-2}. The LT90 at 2000 cd m^{-2} of OLEDs based on p-dPPhen, m-dPPhen and m-dPhen are 31.2 h, 74.5 h and 79.2 h, respectively. The device structure is ITO/HATCN (5 nm)/NPB (30 nm)/BCzPh (10 nm)/*α,β*-ADN:*t*-DABNA (30 nm, 2 wt%)/CzPhPy (10 nm)/DPPyA (20 nm)/EIL (5 nm)/Al.

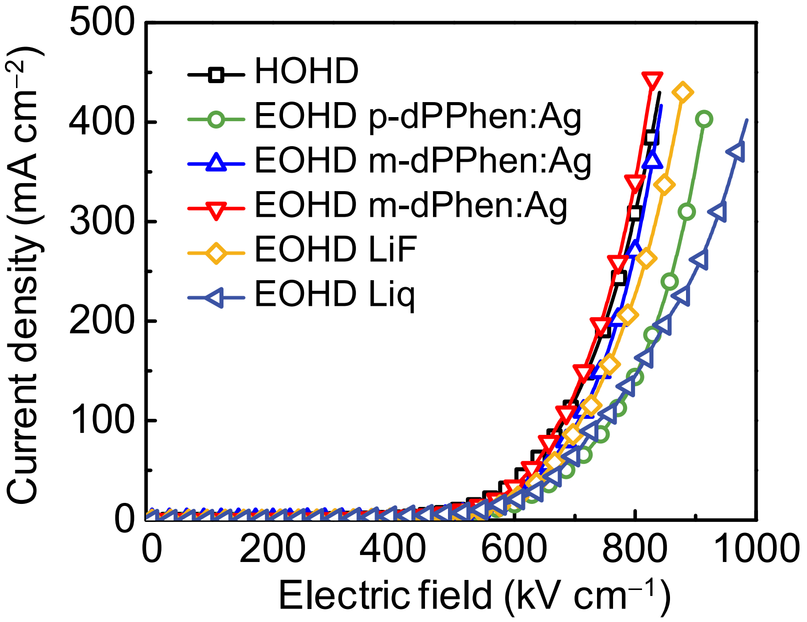

Supplementary Figure 19. The current density–electrical field characteristics of hole-only half-devices (HOHDs) and electron-only half-devices (EOHDs). The device structure of HOHD (black square) is ITO/HATCN (5 nm)/NPB (30 nm)/BCzPh (10 nm)/*α,β*-ADN (30 nm)/Al (150 nm); The device structure of EOHDs are ITO/*α,β*-ADN (30 nm)/CzPhPy (10 nm)/DPPyA (30 nm)/EIL (1 nm for LiF and Liq, 5 nm for Ag-doped p-dPPhen, m-dPPhen and m-dPhen)/Al (150 nm).

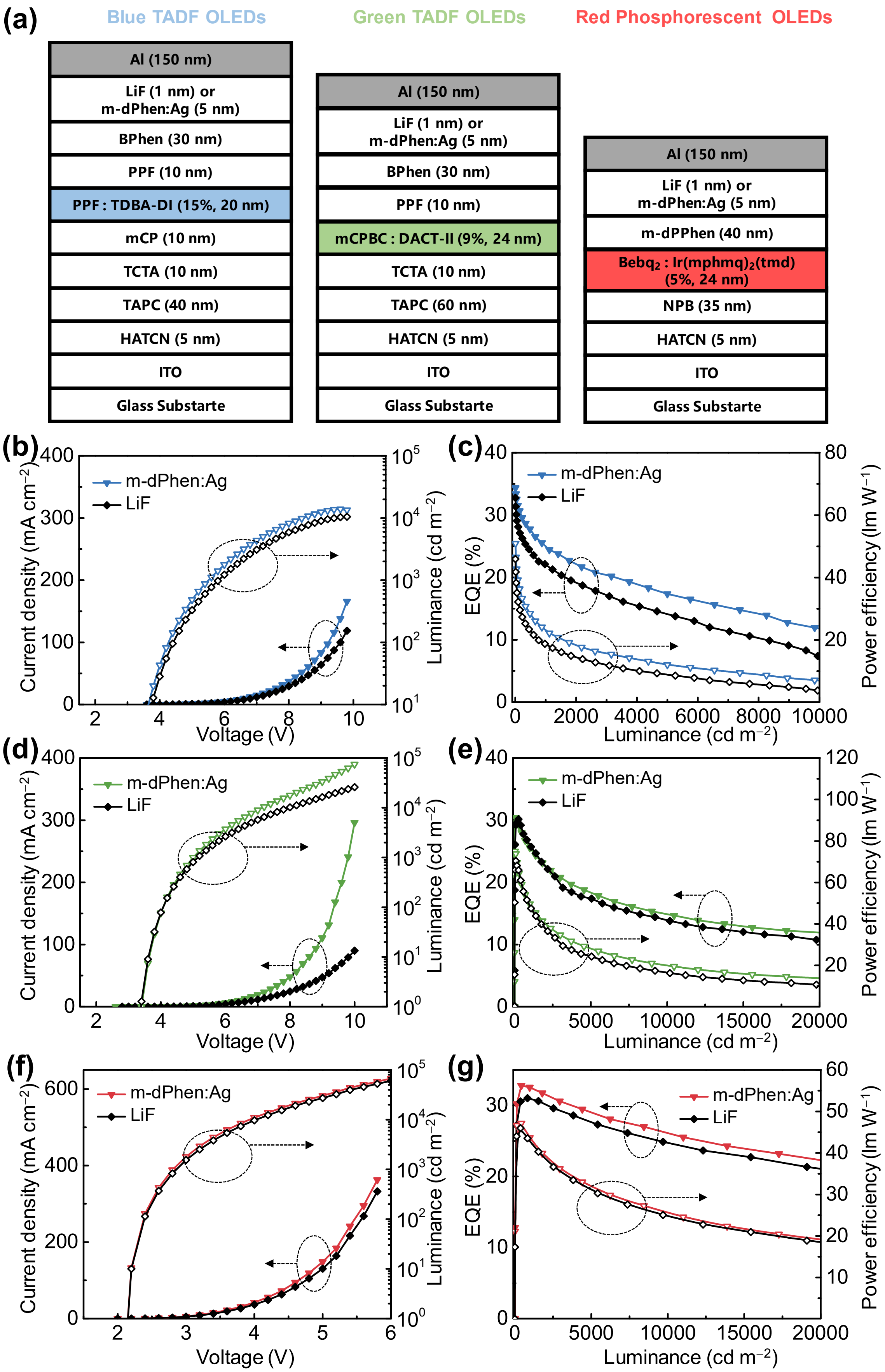

Supplementary Figure 20. (a) The devices structures of blue, green and red OLEDs based on different EILs of LiF (1 nm) or m-dPhen:Ag (5 nm). (b) The current density–voltage–luminance (J–V–L) and (c) the

EQE–power efficiency–luminance curves of blue TADF OLEDs. (d) The $J–V–L$ and (e) the EQE–power efficiency–luminance curves of green TADF OLEDs. (f) The $J-V-L$ and (g) the EQE–power efficiency–luminance curves of red phosphorescent OLEDs.

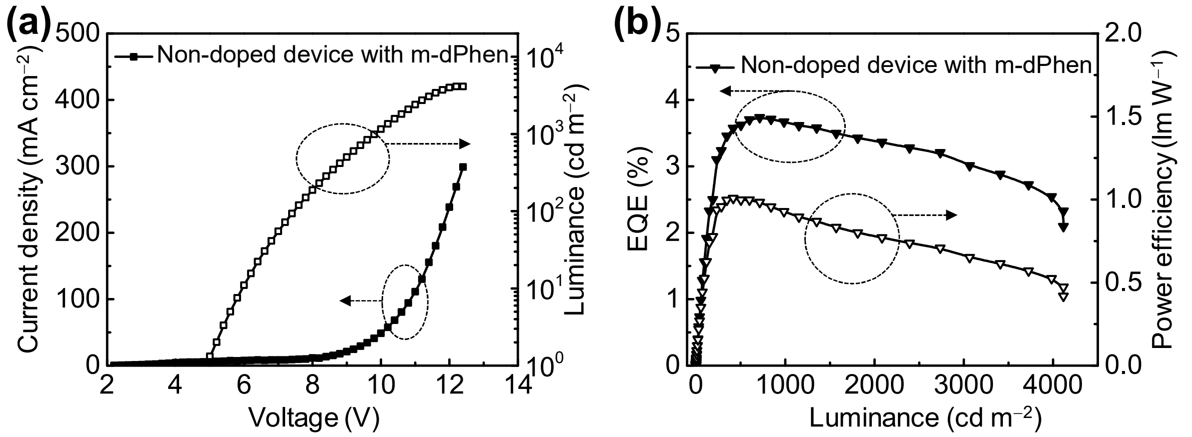

Supplementary Figure 21. (a) The $J–V–L$ of OLED devices with EIL of non-doped m-dPhen (5 nm). (b) The EQE–power efficiency–luminance of OLED devices with EIL of non-doped m-dPhen (5 nm).

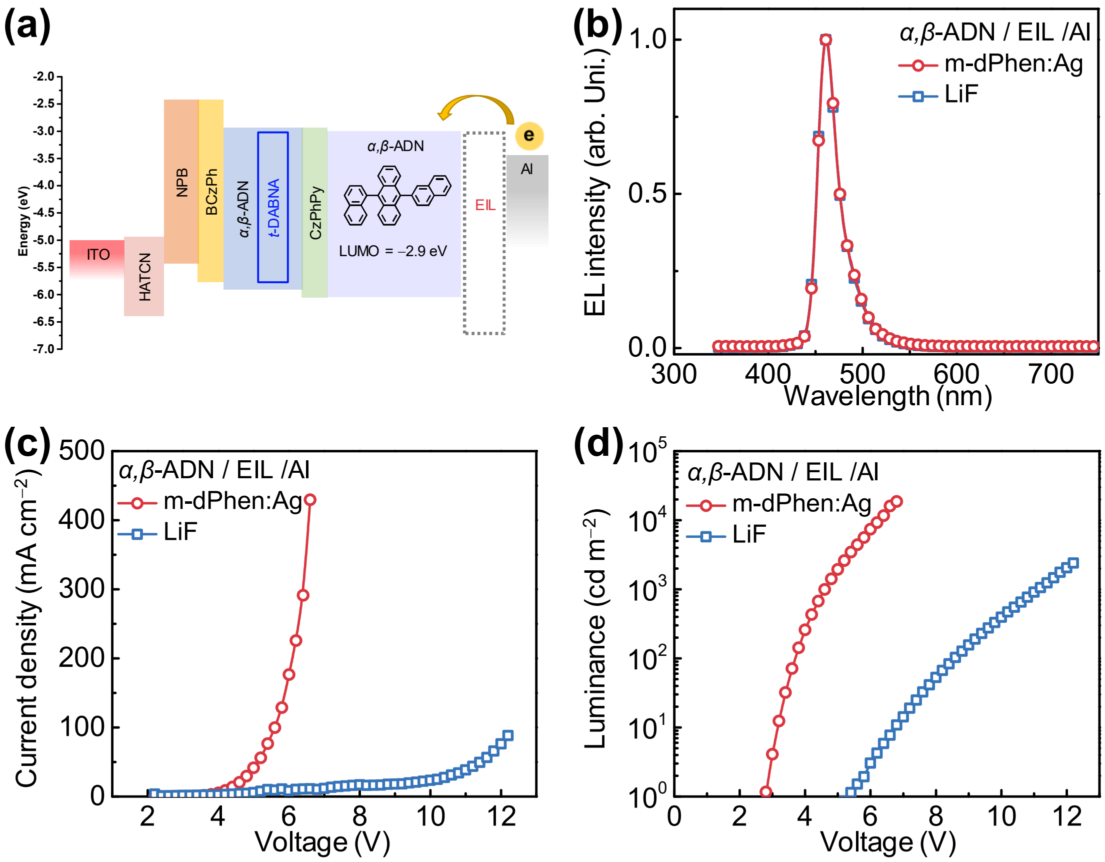

Supplementary Figure 22. (a) Deep-blue OLEDs using PAH-type *α,β*-ADN with LUMO of −2.9 eV as electron transporting materials. (b) Normalized EL spectra at a brightness about 1000 cd m^{-2}. (c) The current density–voltage of OLED devices with ETM of *α,β*-ADN and different EILs. (d) The luminance–voltage of OLED devices with ETM of *α,β*-ADN and different EILs. The device structure is ITO/HATCN (5 nm)/NPB (30 nm)/BCzPh (10 nm)/*α,β*-ADN:*t*-DABNA (30 nm, 2 wt%)/CzPhPy (10 nm)/*α,β*-ADN (20 nm)/EIL/Al. For the device implementing nitrogen-free *α,β*-ADN as ETMs, electroluminescence performances of OLEDs based on m-dPhen:Ag is significantly better than those with LiF, which agrees well with the electron injection efficiencies of m-dPhen:Ag and LiF of EODs in Supplementary Fig. 14.

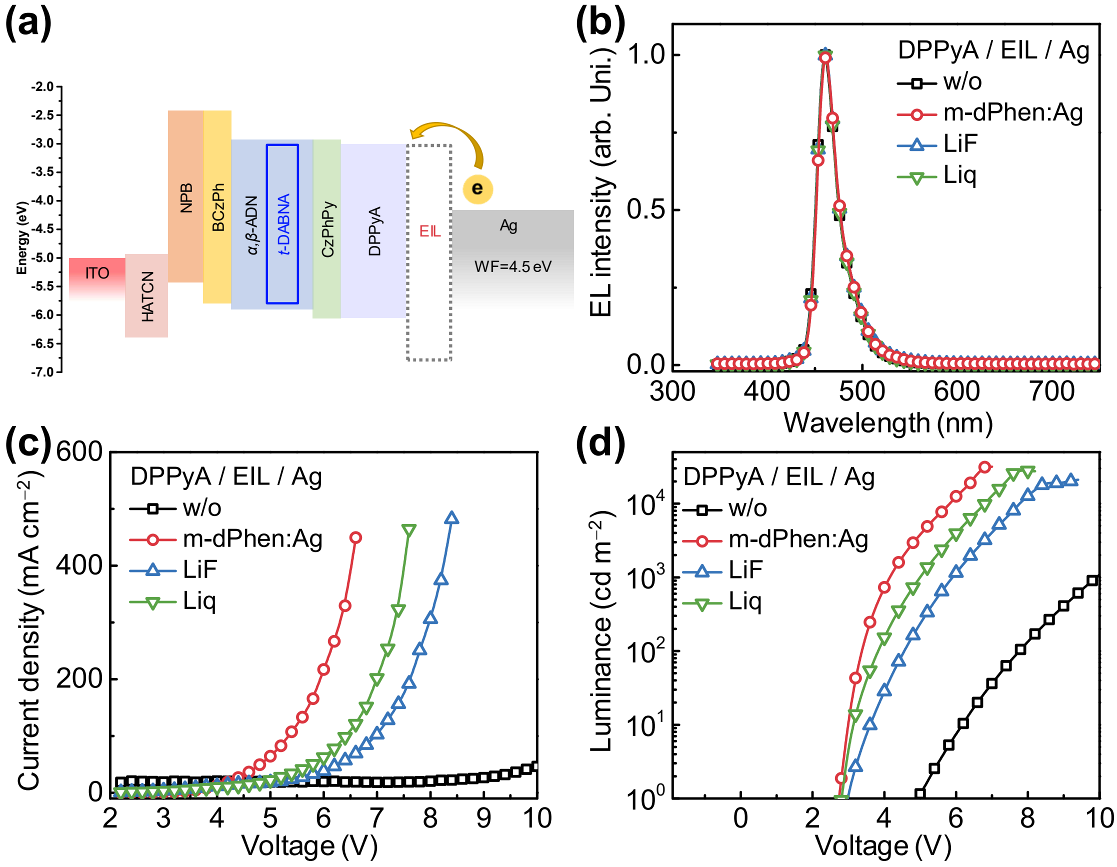

Supplementary Figure 23. (a) Deep-blue OLEDs using silver with high work function of 4.5 eV as cathode. (b) Normalized EL spectra at a brightness about 1000 cd m^{-2}. (c) The current density–voltage of OLED devices with cathode of Ag and different EILs. (d) The luminance–voltage of OLED devices with cathode of Ag and different EILs. The device structure is ITO/HATCN (5 nm)/NPB (30 nm)/BCzPh (10 nm)/*α,β*-ADN:*t*-DABNA (30 nm, 2 wt%)/CzPhPy (10 nm)/DPPyA (20 nm)/EIL/Ag. As plotted in Supplementary Fig. 23, the injection properties of Liq and LiF with silver cathodes are inferior to m-dPhen:Ag. This maybe result from the insufficient coordination reaction at the interface of DPPyA/lithium compound/Ag. Differently, the Ag cathode modified by 5 nm of m-dPhen:Ag (10 wt%) shows a low work function about 3.5 eV, thus enables efficient electron injection.

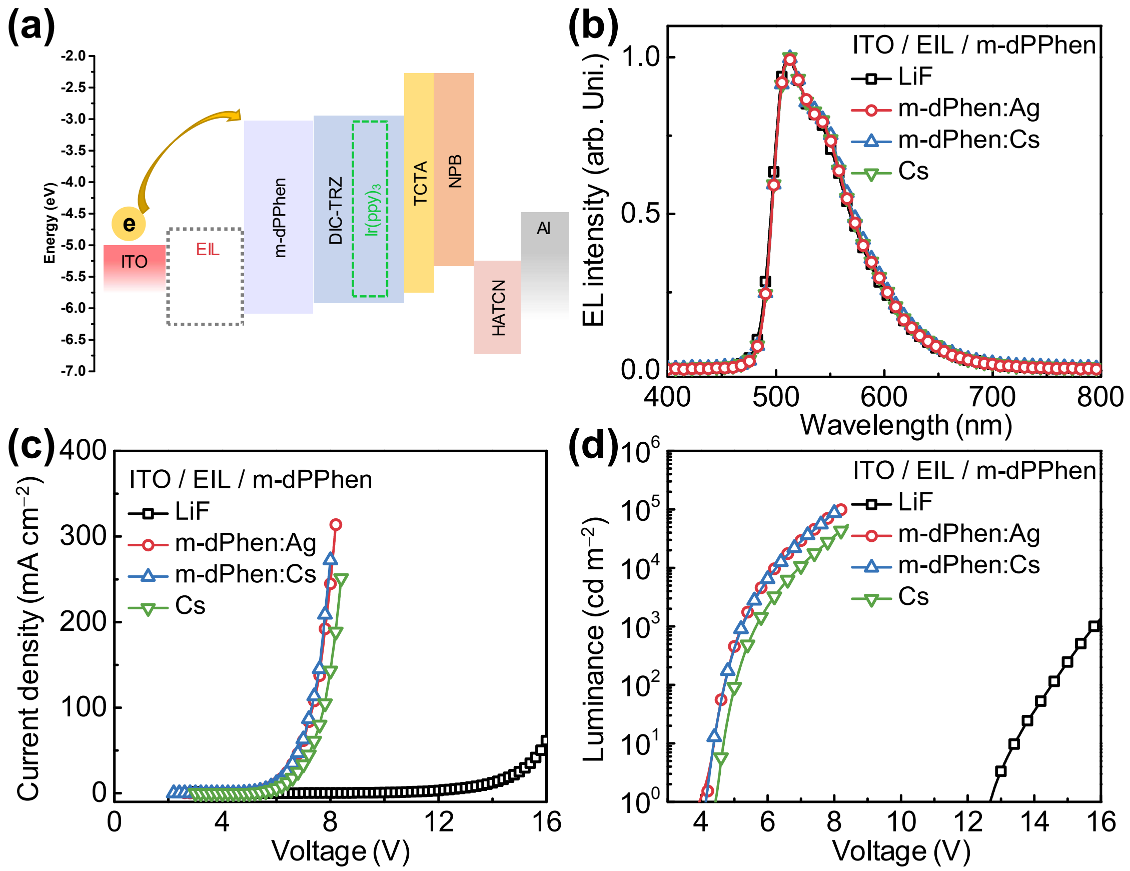

Supplementary Figure 24. (a) Inverted green OLEDs with ITO as cathodes. (b) Normalized EL spectra at the luminance about 1000 cd m^{-2}. (c) The current density–voltage of inverted OLED devices with different EILs. (d) The luminance–voltage of inverted OLED devices with different EILs. The device structure is ITO/LiF(1 nm), m-dPhen:Ag (10 nm, 10 wt%), m-dPhen:Cs (10 nm, 5 wt%) or Cs (1 nm)/m-dPPhen (20 nm)/DIC-TRZ:Ir(ppy)$_3$ (25 nm, 7 wt%)/TCTA (10 nm)/NPB (30 nm)/HATCN (10 nm)/Al (150 nm). The results indicates that m-dPhen:Ag can also function as efficient EIL for ITO cathodes in inverted OLEDs.

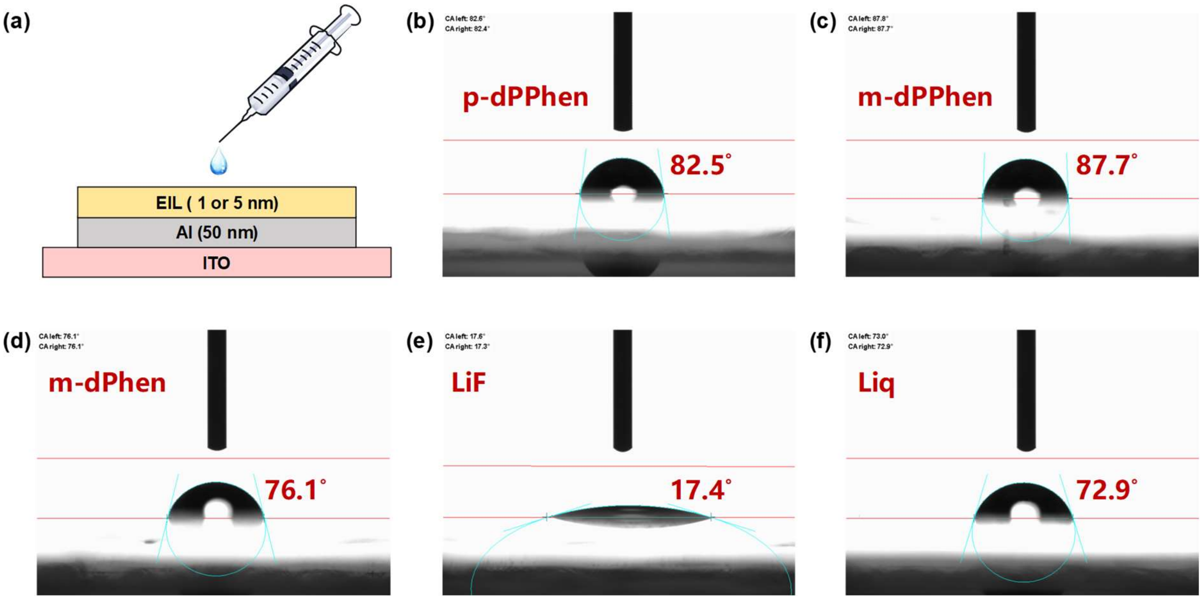

Supplementary Figure 25. (a) The scheme of stable water contact angles on different EIL surfaces. (b) ITO/Al (50 nm)/p-dPPhen:Ag (5 nm, 10%). (c) ITO/Al (50 nm)/m-dPPhen:Ag (5 nm, 10%). (d) ITO/Al (50 nm)/m-dPhen:Ag (5 nm, 10%). (e) ITO/Al (50 nm)/LiF (1 nm). (f) ITO/Al (50 nm)/Liq (1 nm).

Supplementary Figure 26. Single crystal structure with probability ellipsoids of p-dPPhen

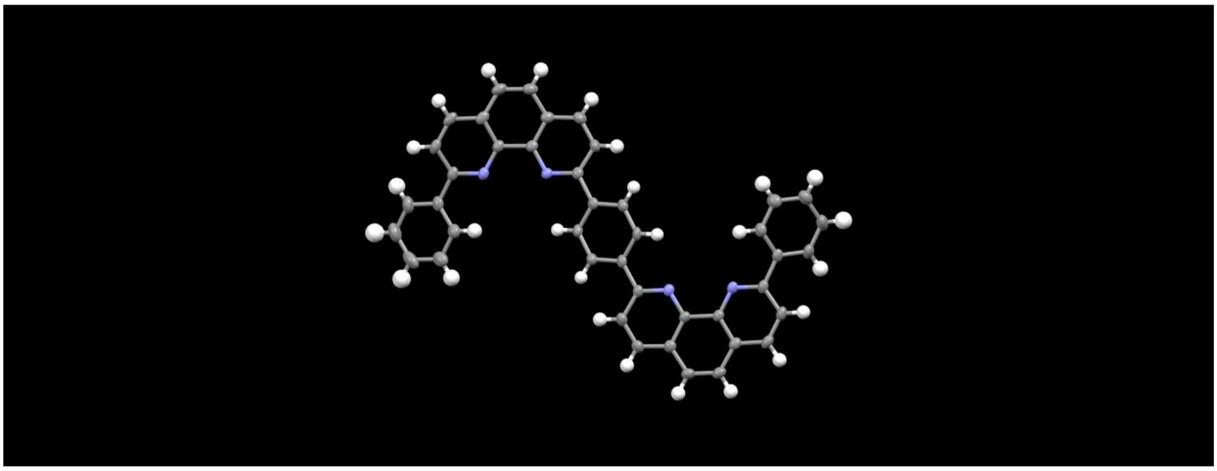

Supplementary Figure 27. Single crystal structure with probability ellipsoids of m-dPPhen.

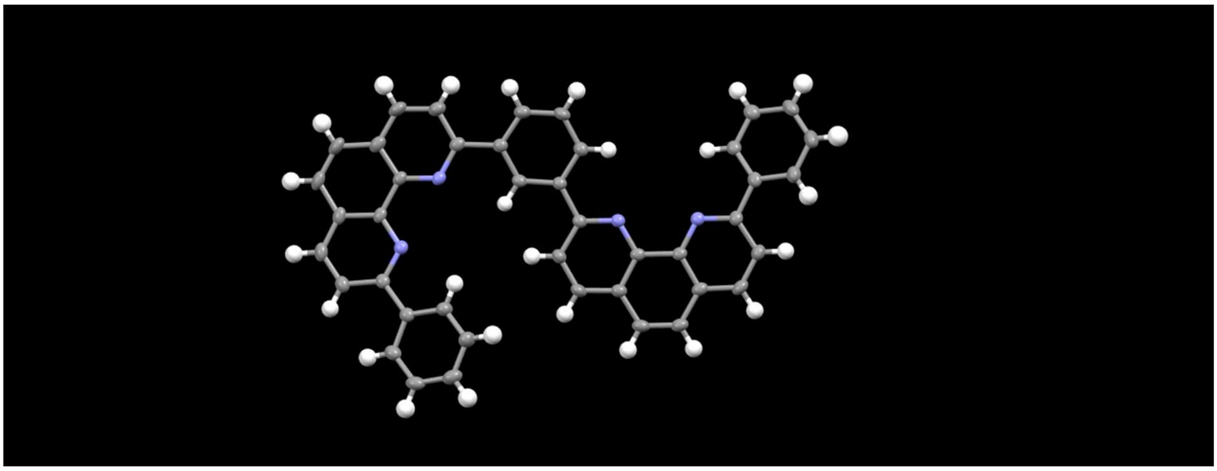

Supplementary Figure 28. Single crystal structure with probability ellipsoids of $[Ag_2(\text{m-dPPhen})_2](NO_3)_2$

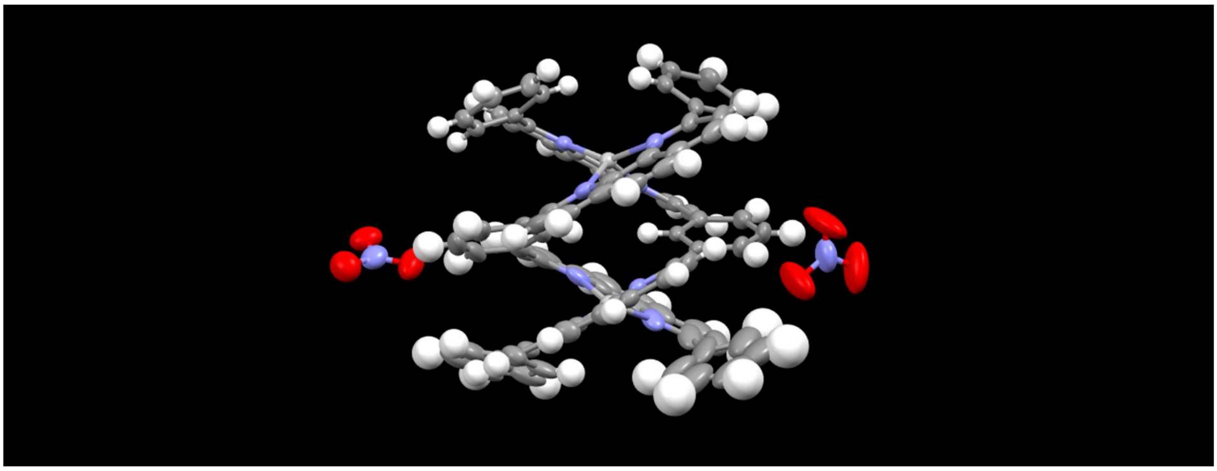

Supplementary Figure 29. Single crystal structure with probability ellipsoids of m-dPhen (solvent evaporation, solvents are omitted).

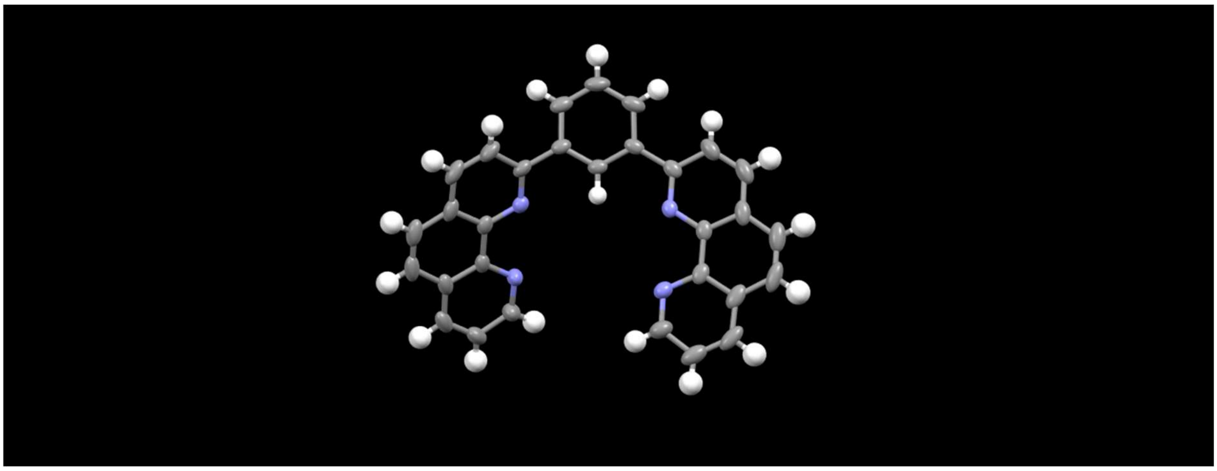

Supplementary Figure 30. Single crystal structure with probability ellipsoids of m-dPhen (sublimation).

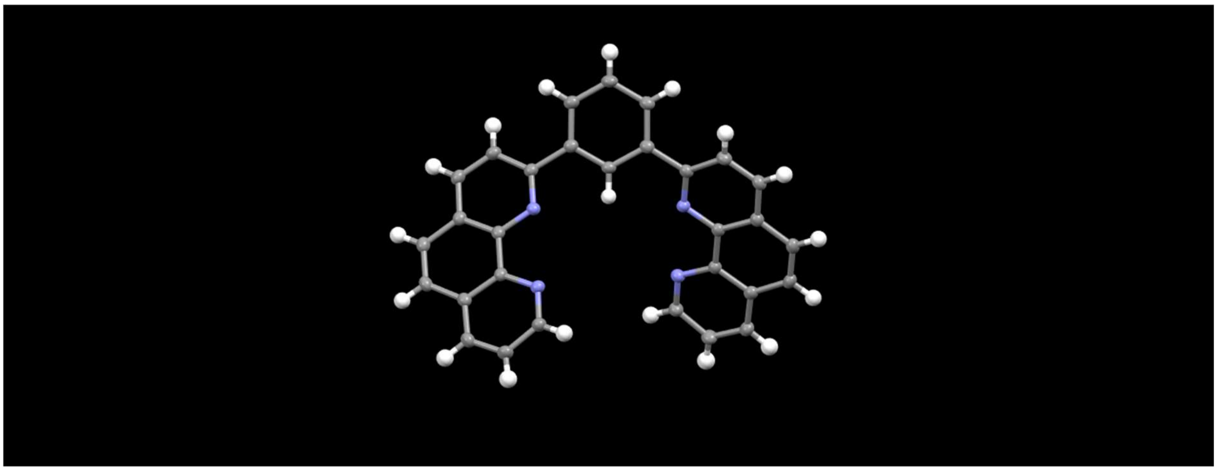

2. Supplementary Tables 1–7

Supplementary Table 1. The physical properties of p-dPPhen, m-dPPhen and m-dPhen

	E_g[a] (eV)	E_{HOMO}[b] (eV)	E_{LUMO}[c] (eV)	S_1[a] (eV)	T_1[a] (eV)	T_g (°C)	T_d (°C)[d]
p-dPPhen	3.18	−6.28	−3.10	3.18	2.34	150	469
m-dPPhen	3.37	−6.30	−2.93	3.22	2.43	138	451
m-dPhen	3.44	−6.30	−2.86	3.41	2.53	136	392

[a] Measured in the toluene solution with a concentration of 10^{-5} mol/L; [b] $E_{HOMO} = E_{LUMO} - E_{opt}$; [c] Measured by using CV[6], $E_{LUMO} = -(E_{re} - E_{Fc/Fc+})$ eV + (−4.8) eV; [d] Decomposition temperature at 5% weight loss.

Supplementary Table 2. Summary of the performances of deep-blue OLEDs based on EILs consisting of Ag-doped diphenanthroline ligands in Supplementary Fig. 18

EIL	Voltage[a] (V)	EQE $_{max/1000/5000}$[b] (%)	PE $_{max/1000/5000}$[b] (lm W^{-1})	CIE (x, y)	Lifetime LT90[c] (h)
p-dPPhen:Ag	4.8	5.3/4.6/5.1	2.2/2.2/2.0	(0.132, 0.085)	31.2
m-dPPhen:Ag	4.4	7.8/7.6/6.9	5.1/4.1/2.9	(0.132, 0.084)	74.5
m-dPhen:Ag	4.2	10.3/10.3/9.5	6.6/5.8/4.4	(0.133, 0.085)	79.2

[a] Values at 1000 cd m^{-2}; [b] Maximum, at 1000 cd m^{-2}, at 5000 cd m^{-2}; [c] Lifetime up to 90% of initial brightness at 2000 cd m^{-2}.

Supplementary Table 3. Summary of the performances of blue, green and red OLEDs based on different EILs of LiF (1 nm) or m-dPhen:Ag (5 nm) in Supplementary Fig. 20

EIL	Emitting dopants	Voltage[a] (V)	EQE $_{max/1000/5000}$[b] (%)	PE $_{max/1000/5000}$[b] (lm W^{-1})
LiF	TDBA-DI	5.8	32.8/22.1/13.8	46.0/18.8/8.6
m-dPhen:Ag	TDBA-DI	5.6	34.4/24.4/17.4	50.9/22.2/12.1
LiF	DACT-II	5.2	30.2/25.7/17.4	70.4/47.4/24.3
m-dPhen:Ag	DACT-II	5.0	30.4/25.6/17.9	73.8/49.6/26.9
LiF	$Ir(mphmq)_2(tmd)$	3.0	31.0/30.6/27.3	46.0/40.3/30.3
m-dPhen:Ag	$Ir(mphmq)_2(tmd)$	2.8	32.8/32.5/28.1	47.1/43.4/29.8

[a] Values at 1000 cd m^{-2}; [b] Maximum, at 1000 cd m^{-2}, at 5000 cd m^{-2}.

Supplementary Table 4. Summary of the performances of deep-blue OLEDs (bottom-emitting) based on triplet-triplet annihilation (TTA).

Ref.	Voltage[a)] (V)	Voltage[b)] (V)	EQE[c)] (%)	EQE[b)] (%)	CIE (x, y)	Lifetime LT90[d)] (h)
1	N.A.	N.A.	> 9	N.A.	N.A.	N.A.
7	2.9	N.A.	7.9	7.9	(0.13, 0.14)	N.A.
8	2.5	3.4	9.8	9.8	(0.15, 0.21)	N.A.
9	N.A.	N.A.	7.6	7.0	(0.126, 0.098)	608 h @ 200 cd m^{-2}
10	2.9	4.5	9.36	9.18	(0.134, 0.115)	N.A.
11	N.A.	N.A.	7.4	N.A.	(0.15, 0.22)	N.A.
12	3.2	N.A.	5.11	5.10	(0.154, 0.141)	N.A.
**This work**	**2.8**	**4.2**	**10.3**	**10.3**	**(0.133, 0.085)**	**79.2 h @ 2000 cd m^{-2}**

[a)] Values at 1 cd m^{-2}; [b)] Values at 1000 cd m^{-2}; [c)] Maxmium values; [d)] Time to 90% of the initial luminance; N.A. The values were not assigned.

Supplementary Table 5. The color coordinate corrected current efficiency (CCE) values and the lifetimes of deep-blue devices with a y color coordinate < 0.2.

Ref.	CIE y	CCE[a)] (cd/A/y)	CCE[b)] (cd/A/y)	Lifetime (h)
7	0.14	64.3	64.3	N.A.
9	0.098	63.3	59.2	T90=608 h @ 200 cd m^{-2}
10	0.115	68.2	67.1	N.A.
12	0.141	41.7	N.A.	N.A.
13	0.09	121.1	N.A.	T50=17 h @ 200 cd m^{-2}
13	0.115	203.5	N.A.	T50=293 h @ 200 cd m^{-2}
14	0.06	141.8	63.3	N.A.
14	0.15	233.8	192.9	N.A.
15	0.11	283.6	210.9	N.A.
16	0.18	165.6	N.A.	N.A.
17	0.13	223.8	N.A.	T50=10700 h @ 100 cd m^{-2}
18	0.19	94.7	40.5	N.A.
19	0.18	151.7	N.A.	N.A.
19	0.11	117.3	N.A.	N.A.
19	0.15	122.0	N.A.	N.A.
20	0.20	189.0	162.5	N.A.
20	0.08	107.5	45.0	N.A.
21	0.09	364	262.2	T50=117 h @ 1000 cd m^{-2}
21	0.09	432	271.1	T50=97 h @ 1000 cd m^{-2}
22	0.18	208.9	N.A.	N.A.
23	0.15	162.7	55.3	N.A.
24	0.19	58.4	N.A.	N.A.
**This work**	**0.045**	**237.3**	**236.8**	**T95=104.1 h @ 5000 cd m^{-2}**

[a)] Maxmium values; [b)] Values at 1000 cd m^{-2}; N.A. The values were not assigned.

Supplementary Table 6. X-ray crystallographic data and structure refinement for p-dPPhen, m-dPPhen and $[Ag_2(m\text{-dPPhen})_2](NO_3)_2$.

Sample	p-dPPhen	m-dPPhen	$[Ag_2(m\text{-dPPhen})_2](NO_3)_2$
Empirical formula	C42 H26 N4	C44 H32 Cl4 N4 O	C84 H52 Ag2 N10 O6
Formula weight	586.67	774.53	1513.09
Temperature	165.15 K	153.15 K	100.00(10) K
Wavelength	0.71073 Å	0.71073 Å	1.54184 Å
Crystal system	Trigonal	Monoclinic	Tetragonal
Space group	R -3	C 1 2/c 1	$I4_1/a$
Unit cell dimensions	a = 35.472(5) Å<br>b = 35.472(5) Å<br>c = 12.264(3) Å<br>a= 90°<br>b= 90°<br>g = 120°	a = 31.674(6) Å<br>b = 12.338(3) Å<br>c = 21.599(4) Å<br>a= 90°<br>b= 119.14(3)°<br>g = 90°	a = 44.1581(4) Å<br>b = 44.1581(4) Å<br>c = 15.7741(2) Å<br>a= 90°<br>b= 90°<br>g = 90°
Volume	13363(5) $Å^3$	7373(3) $Å^3$	30758.6(6) $Å^3$
Z	18	8	16
Density (calculated)	1.312 Mg m^{-3}	1.396 Mg m^{-3}	1.307 Mg m^{-3}
Absorption coefficient	0.078 mm^{-1}	0.363 mm^{-1}	4.554 mm^{-1}
F(000)	5508	3200	12288
Theta range for data collection	2.125 to 24.998°	2.539 to 27.486°	3.586 to 75.964°
Index ranges	-42<=h<=42<br>-42<=k<=42<br>-14<=l<=14	-41<=h<=41<br>-15<=k<=16<br>-24<=l<=28	-50<=h<=55<br>-44<=k<=48<br>-14<=l<=19
Reflections collected	46654	24545	47738
Independent reflections	5213 [R(int) = 0.0654]	8363 [R(int) = 0.0423]	15350 [R(int) = 0.0388]
Absorption correction	Semi-empirical from equivalents		
Max/min transmission	1.0000 and 0.8649	1.0000 and 0.8215	1.00000 and 0.42322
Refinement method	Full-matrix least-squares on F^2		
Data/restraints/parameters	5213 / 0 / 415	8363 / 72 / 506	15350 / 90 / 907
Goodness-of-fit on F^2	1.364	1.164	1.055
Final R indices [I>2sigma(I)]	R1 = 0.0818<br>wR2 = 0.1536	R1 = 0.0703<br>wR2 = 0.1467	R1 = 0.0556<br>wR2 = 0.1461
R indices (all data)	R1 = 0.0887<br>wR2 = 0.1567	R1 = 0.0789<br>wR2 = 0.1518	R1 = 0.0639<br>wR2 = 0.1515
Extinction coefficient	n/a	n/a	n/a
Largest diff. peak and hole ($e.Å^{-3}$)	0.215 and -0.177 $e.Å^{-3}$	0.401 and -0.541 $e.Å^{-3}$	1.522 and -0.647 $e.Å^{-3}$

Supplementary Table 7. X-ray crystallographic data and structure refinement for m-dPhen obtained from solvent evaporation and sublimation.

Sample	m-dPhen·CH_2Cl_2<br>(solvent evaporation)	m-dPhen<br>(sublimation)
Empirical formula	C31 H20 Cl2 N4	C30 H18 N4
Formula weight	519.41	434.48
Temperature	163.15 K	99.99(10) K
Wavelength	0.71073 Å	1.54184 Å
Crystal system	Orthorhombic	Orthorhombic
Space group	Pbca	Pbcn
Unit cell dimensions	a = 16.915(3) Å<br>b = 9.4251(19) Å<br>c = 31.043(6) Å<br>a= 90°<br>b= 90°<br>g = 90°	a = 19.1857(5) Å<br>b = 8.4846(2) Å<br>c = 12.8401(3) Å<br>a= 90°<br>b= 90°<br>g = 90°
Volume	4948.9(17) Å^3	2090.15(9) Å^3
Z	8	4
Density (calculated)	1.394 Mg m^{-3}	1.381 Mg m^{-3}
Absorption coefficient	0.292 mm^{-1}	0.651 mm^{-1}
F(000)	2144	904
Theta range for data collection	1.312 to 25.000°	4.609 to 76.365°.
Index ranges	-18<=h<=20<br>-11<=k<=7<br>-36<=l<=25	-19<=h<=24<br>-10<=k<=3<br>-12<=l<=16
Reflections collected	15979	7374
Independent reflections	4335 [R(int) = 0.0497]	2086 [R(int) = 0.0428]
Absorption correction	Semi-empirical from equivalents	
Max/min transmission	1.0000 and 0.7043	1.00000 and 0.54415
Refinement method	Full-matrix least-squares on F^2	
Data/restraints/parameters	4335 / 474 / 334	2086 / 0 / 156
Goodness-of-fit on F^2	1.212	1.087
Final R indices [I>2sigma(I)]	R1 = 0.0760<br>wR2 = 0.1439	R1 = 0.0493<br>wR2 = 0.1366
R indices (all data)	R1 = 0.0920<br>wR2 = 0.1507	R1 = 0.0539<br>wR2 = 0.1404
Extinction coefficient	n/a	0.0020(5)
Largest diff. peak and hole (e.Å^{-3})	0.271 and -0.232 e.Å^{-3}	0.255 and -0.245 e.Å^{-3}

3. Supplementary Methods

3.1 General information

All of the synthesized organic materials are purified by train sublimation before the measurement and device fabrication. The molecular structures of synthsized materials are indentified by ^{1}H NMR spectra with JEOLAL-600 MHz spectrometer at ambient atmosphere with tetramethylsilane (TMS) as the internal standard. MALDI-TOF MS spectra was measured on a Performance (Shimadzu, Japan). The ultraviolet (UV)-vis absorption spectra were recorded by an Agilent 8453 spectrophotometer. The PL spectra of solution samples are recorded by Hitachi F-7000 spectrometer.

3.2 Thermal properties measurements

The thermogravimetric analysis (TGA) was recorded on a STA 409PC thermogravimeter at a heating rate of 10 °C min^{-1} from room temperature to 600 °C under nitrogen atmosphere. The differential scanning calorimetry (DSC) measurements were measured on DSC 2910 modulated calorimeter at a heating rate of 10 °C min^{-1} from room temperature to decomposition temperature under a nitrogen atmosphere.

3.3 Electrochemical Measurement

The electrochemical measurement were conducted on an electrochemical analyzer (CHI600E, CH Instruments Ins.) by employing Ag/AgCl electrode as reference and platinum (Pt) as working electrodes with Pt wire as auxiliary electrode. The LUMO levels of diphenanroline derivatives are obtained by the reduction potentials against ferrocene/ferrocenium. The HOMO levels of designed materials are estimated by LUMO levels and optical energy gaps (E_gs).

3.4 Synthesis of diphenanthroline derivatives

Synthesis of 1,4-bis(9-phenyl-1,10-phenanthrolin-2-yl)benzene (p-dPPhen)

1,4-bis(4,4,5,5-tetramethyl-1,3,2-dioxaborolan-2-yl)benzene (2.80 g, 8.48 mmol) and 2-bromo-9-phenyl-1,10-phenanthroline (6.43 g, 19.17 mmol, 2.26 equiv.) and $Pd(PPh_3)_4$ (1.08 g, 0.933 mmol, 0.11 equiv.) and Na_2CO_3 (6.12 g, 57.78 mmol, 6.81 equiv.) were added to the mixed solvent of toluene/EtOH/H_2O with 150/50/100 mL) in a two-necked flask equipped with a magnetic stirrer. The mixture are allowed to reflux at 100 °C for 30 h under nitrogen atmosphere. The resulting mixture was cooled down to room temperature and

filted to collect precipitate. The precipitate are sequentially washed with deionized water, methanol and methylene chloride. The resulting residue was further purified by train sublimation to afford p-dPPhen.

^{1}H NMR (600 MHz, $CDCl_3$) δ/ppm: 8.74 (s, 4H), 8.52 (d, 4H), 8.38 (dd, 4H), 8.32 (dd, 2H), 8.20 (d, 2H), 7.84 (s, 4H), 7.61 (t, 4H), 7.52 (t, 2H). MS (MALDI-TOF) m/z: 587.29 $[M+H]^+$.

Synthesis of 1,3-di(1,10-phenanthrolin-2-yl)benzene (m-dPhen)

1,3-bis(4,4,5,5-tetramethyl-1,3,2-dioxaborolan-2-yl)benzene (2.80 g, 8.48 mmol) and 2-chloro-1,10-phenanthroline (4.12 g, 19.17 mmol, 2.26 equiv.) and $Pd(PPh_3)_4$ (1.08 g, 0.933 mmol, 0.11 equiv.) and Na_2CO_3 (6.12 g, 57.78 mmol, 6.81 equiv.) were added to the mixed solvent of toluene/EtOH/H_2O with 150/50/100 mL) in a two-necked flask equipped with a magnetic stirrer. The mixture are allowed to reflux at 100 °C for 30 h under nitrogen atmosphere. The resulting mixture was cooled down to room temperature and extracted with 500 mL of methylene chloride by two times. The organic layers are dried by Na_2SO_4, filtered and evaporated to remove extra solvent. The resulting crude products was further purified column chromatography and followed by train subimation to afford m-dPhen.

^{1}H NMR (600 MHz, $CDCl_3$) δ/ppm: 9.25 (dd, 2H), 9.14 (s, 1H), 8.54 (d, 2H), 8.35 (m, 4H), 8.27 (d, 2H), 7.82 (m, 4H), 7.74 (t, 1H), 7.65 (dd, 2H). MS (MALDI-TOF) m/z: 435.24 $[M+H]^+$.

Synthesis of 1,3-bis(9-phenyl-1,10-phenanthrolin-2-yl)benzene (m-dPPhen)

1,3-bis(4,4,5,5-tetramethyl-1,3,2-dioxaborolan-2-yl)benzene (2.80 g, 8.48 mmol) and 2-bromo-9-phenyl-1,10-phenanthroline (6.43 g, 19.17 mmol, 2.26 equiv.) and $Pd(PPh_3)_4$ (1.08 g, 0.933 mmol, 0.11 equiv.) and Na_2CO_3 (6.12 g, 57.78 mmol, 6.81 equiv.) were added to the mixed solvent of toluene/EtOH/H_2O with 150/50/100 mL) in a two-necked flask equipped with a magnetic stirrer. The mixture are allowed to reflux at 100 °C for 30 h under nitrogen atmosphere. The resulting mixture was cooled down to room temperature and filted to collect precipitate. The precipitate are sequentially washed with deionized water, methanol and methylene chloride. The resulting residue was further purified by train subimation to afford m-dPPhen.

^{1}H NMR (400 MHz, $CDCl_3$) δ/ppm: 9.74 (s, 1H), 8.72 (dd, 2H), 8.54 (m, 4H), 8.50 (d, 2H), 8.42 (d, 2H), 8.35 (d, 2H), 8.18 (d, 2H), 7.86 (m, 5H), 7.58 (t, 4H), 7.52 (t, 2H). MS (MALDI-TOF) m/z: 587.33 $[M+H]^+$.

4. Supplementary Reference

[1] Fukagawa, H. et al. Understanding coordination reaction for producing stable electrode with various low work functions. *Nat. Commun.* 11, 3700 (2021).

[2] Fukagawa, H., Hasegawa, M., Morii, K., Suzuki, K., Sasaki, T. & Shimizu, T. Universal Strategy for Efficient Electron Injection into Organic Semiconductors Utilizing Hydrogen Bonds. *Adv. Mater.* 31, 1904201 (2019).

[3] Dietrich-Buchecker, C., Colasson, B., Jouvenot, D. & Sauvage, J. -P. Synthesis of multi-1,10-phenanthroline ligands with 1,3-phenylene linkers and their lithium complexes. *Chem.-Eur. J.* 11, 4374-4386 (2005).

[4] Rapenne, G., Dietrich-Buchecker, C. & Sauvage, J. -P. Copper (I)- or Iron (II)-Templated Synthesis of Molecular Knots Containing Two Tetradedral or Octahedral Coordination Sites. *J. Am. Chem. Soc.* 121, 994-1001 (1999).

[5] Sun, Y. et al. A Pyridine-Containing Anthracene Derivative with High Electron and Hole Mobilities for Highly Efficient and Stable Fluorescent Organic Light-Emitting Diodes. *Adv. Funct. Mater.* 21, 1881-1886 (2011).

[6] Bian, M. et al. Long-Lived and Highly Efficient TADF-PhOLED with "(A)n–D–(A)n" Structured Terpyridine Electron Transporting Material. *Adv. Funct. Mater.* 28, 1800429 (2018).

[7] Lim, H. et al. Enhanced Triplet-Triplet Annihilation of Blue Fluorescent Organic Light Emitting Diodes by Generating Excitons in Trapped Charge-Free Regions. *ACS Appl. Mater. Interfaces* 11, 48121–48127 (2019).

[8] Salehi, A. et al. Realization of high-efficiency fluorescent organic light-emitting diodes with low driving voltage. *Nat. Commun.* 10, 2305 (2019).

[9] Lee, K. H. & Lee, J. Y. Paradigm change of blue emitters: Thermally activated fluorescence emitters as long-living fluorescence emitters by triplet exciton quenching. *Org. Electron.* 75, 105377 (2019).

[10] Bae, H. W. et al. Efficiency enhancement in fluorescent deep-blue OLEDs by boosting singlet exciton generation through triplet fusion and charge recombination rate. *Org. Electron.* 70, 1-6 (2019).

[11] Fukagawa, H. et al. Anthracene derivatives as efficient emitting hosts for blue organic light-emitting diodes utilizing triplet–triplet annihilation. *Org. Electron.* 13, 1197-1203 (2012).

[12] Shan, T. et al. Highly efficient and stable pure blue nondoped organic light-emitting diodes at high luminance based on phenanthroimidazole-pyrene derivative enabled by triplei-triplet annihilation. *Dyes Pigment.* 142, 189-197 (2017).

[13] Lee, K. H. & Lee, J. Y. Phosphor sensitized thermally activated delayed fluorescence organic light-emitting diodes with ideal deep blue device performances. *J. Mater. Chem. C* 7, 8562-8568 (2019).

[14] Ahn, D. H. et al. Highly efficient blue thermally activated delayed fluorescence emitters based on symmetrical and rigid oxygen-bridged boron acceptors. *Nat. Photon.* 13, 540-546 (2019).

[15] Kondo, Y. et al. Narrowband deep-blue organic light-emitting diode featuring an organoboronbased emitter. *Nat. Photon.* 13, 678-682 (2019).

[16] Kim, J. U. et al. Nanosecond-time-scale delayed fluorescence molecule for deep-blue OLEDs with small efficiency roll off. *Nat. Commun.* 11, 1765 (2020).

[17] Jung, M. N., Lee, K. H., Lee, J. Y. & Kim, T. K. A bipolar host based high triplet energy electroplex for an over 10,000 h lifetime in pure blue phosphorescent organic light-emitting diodes. *Mater. Horiz.* 7, 559-565 (2020).

[18] Kim, H. G., Shin, H., Ha, Y. H., Kim, R., Kwon, S. K., Kim, Y. H. & Kim, J. J. Triplet Harvesting by a Fluorescent Emitter Using a Phosphorescent Sensitizer for Blue OrganicLight-Emitting Diodes. *ACS Appl. Mater. Interfaces* 11, 26-30 (2019).

[19] Ahn, D. H., Jeong, J. H., Song, J., Lee, J. Y. & Kwon, J. H. Highly Efficient Deep Blue Fluorescent Organic Light-Emitting Diodes Boosted by Thermally Activated Delayed Fluorescence Sensitization. *ACS Appl. Mater. Interfaces* 10, 10246–10253 (2018).

[20] Ahn, D. H. et al. Highly Twisted Donor–Acceptor Boron Emitter and High Triplet Host Material for Highly Efficient Blue Thermally Activated Delayed Fluorescent Device. *ACS Appl. Mater. Interfaces* 11, 14909-14916 (2019).

[21] Jeon, S. O. et al. High-efficiency, long-lifetime deep-blue organic light-emitting diodes. *Nat. Photon.* 15, 208-215 (2021).

[22] Rajamalli, P., Senthilkumar, N., Huang, P. Y., Wu, C. C., Lin, H. W. & Cheng, C. H. New Molecular Design Concurrently Providing Superior Pure Blue, Thermally Activated Delayed Fluorescence and Optical Out-Coupling Efficiencies. *J. Am. Chem. Soc.* 139, 10948-10951 (2017).

[23] Liu, M. et al. Horizontally Orientated Sticklike Emitters: Enhancement of Intrinsic Out-Coupling Factor and Electroluminescence Performance. *Chem. Mater.* 29, 8630-8636 (2017).

[24] Kukhta, N. A. et al. Deep-Blue High-Efficiency TTA OLED Using *Para*- and *Meta*- Conjugated Cyanotriphenylbenzene and Carbazole Derivatives as Emitter and Host. *J. Phys. Chem. Lett.* 8, 6199-6205 (2017).
