# Supplementary material for: In situ-formed tetrahedrally coordinated double-helical metal complexes for improved coordination-activated n-doping
Source: Nat Commun. 2022 Mar 8;13:1215. doi: 10.1038/s41467-022-28921-5 (PMC8904628; doi:10.1038/s41467-022-28921-5)
Supplement: Supplementary file 3 — Description of Additional Supplementary Information [file 41467_2022_28921_MOESM3_ESM.pdf]

Description of Additional Supplementary Information

Title: Supplementary Data 1

Description: Crystallographic data and CheckCIF files as .zip files
