# Supplementary material for: In situ-formed tetrahedrally coordinated double-helical metal complexes for improved coordination-activated n-doping
Source: Nat Commun. 2022 Mar 8;13:1215. doi: 10.1038/s41467-022-28921-5 (PMC8904628; doi:10.1038/s41467-022-28921-5)

checkCIF/PLATON report

Structure factors have been supplied for datablock(s) 20200905b

THIS REPORT IS FOR GUIDANCE ONLY. IF USED AS PART OF A REVIEW PROCEDURE FOR PUBLICATION, IT SHOULD NOT REPLACE THE EXPERTISE OF AN EXPERIENCED CRYSTALLOGRAPHIC REFEREE.

No syntax errors found.   CIF dictionary   Interpreting this report

Datablock: 20200905b

Bond precision:	C-C = 0.0079 A		Wavelength=1.54184
Cell:	a=44.1581(4)	b=44.1581(4)	c=15.7741(2)
	alpha=90	beta=90	gamma=90
Temperature:	100 K		

	Calculated	Reported
Volume	30758.5(7)	30758.6(6)
Space group	I 41/a	I 41/a
Hall group	-I 4ad	-I 4ad
Moiety formula	C84 H52 Ag2 N8, 2(N O3) [+ solvent]	C84 H52 Ag2 N8, 2(N O3)
Sum formula	C84 H52 Ag2 N10 O6 [+ solvent]	C84 H52 Ag2 N10 O6
Mr	1513.10	1513.09
Dx,g cm-3	1.307	1.307
Z	16	16
Mu (mm-1)	4.554	4.554
F000	12288.0	12288.0
F000’	12325.12	
h,k,lmax	55,55,19	55,48,19
Nref	16037	15350
Tmin,Tmax	0.608,0.695	0.423,1.000
Tmin’	0.481	

Correction method= # Reported T Limits: Tmin=0.423 Tmax=1.000
AbsCorr = MULTI-SCAN

Data completeness= 0.957   Theta(max)= 75.964

R(reflections)= 0.0556(13216)   wR2(reflections)= 0.1515(15350)

S = 1.055   Npar= 907

The following ALERTS were generated. Each ALERT has the format
test-name_ALERT_alert-type_alert-level.
Click on the hyperlinks for more details of the test.

Alert level C

```
PLAT094_ALERT_2_C Ratio of Maximum / Minimum Residual Density ....       2.35 Report
PLAT220_ALERT_2_C NonSolvent   Resd 1  C   Ueq(max)/Ueq(min) Range        5.3 Ratio
PLAT222_ALERT_3_C NonSolvent Resd 1  H   Uiso(max)/Uiso(min) Range        4.8 Ratio
PLAT230_ALERT_2_C Hirshfeld Test Diff for    C6       --C7       .        6.0 s.u.
PLAT230_ALERT_2_C Hirshfeld Test Diff for    C9       --C10      .        6.0 s.u.
PLAT230_ALERT_2_C Hirshfeld Test Diff for    C27      --C28      .        5.5 s.u.
PLAT230_ALERT_2_C Hirshfeld Test Diff for    C31      --C32      .        6.7 s.u.
PLAT230_ALERT_2_C Hirshfeld Test Diff for    C41      --C42      .        5.9 s.u.
PLAT234_ALERT_4_C Large Hirshfeld Difference C35      --C36      .       0.16 Ang.
PLAT234_ALERT_4_C Large Hirshfeld Difference C36      --C37      .       0.17 Ang.
PLAT234_ALERT_4_C Large Hirshfeld Difference C82      --C83      .       0.16 Ang.
PLAT241_ALERT_2_C High   'MainMol' Ueq as Compared to Neighbors of        C39 Check
PLAT241_ALERT_2_C High   'MainMol' Ueq as Compared to Neighbors of        C41 Check
PLAT242_ALERT_2_C Low    'MainMol' Ueq as Compared to Neighbors of        C42 Check
PLAT244_ALERT_4_C Low    'Solvent' Ueq as Compared to Neighbors of         N9 Check
PLAT244_ALERT_4_C Low    'Solvent' Ueq as Compared to Neighbors of        N10 Check
PLAT260_ALERT_2_C Large Average Ueq of Residue Including        O4      0.146 Check
PLAT911_ALERT_3_C Missing FCF Refl Between Thmin & STh/L=    0.600         72 Report
PLAT977_ALERT_2_C Check Negative Difference Density on H39              -0.31 eA-3
PLAT977_ALERT_2_C Check Negative Difference Density on H40              -0.33 eA-3
```

Alert level G

```
PLAT003_ALERT_2_G Number of Uiso or Uij Restrained non-H Atoms ...         15 Report
PLAT083_ALERT_2_G SHELXL Second Parameter in WGHT  Unusually Large     108.23 Why ?
PLAT186_ALERT_4_G The CIF-Embedded .res File Contains ISOR Records          2 Report
PLAT333_ALERT_2_G Large Aver C6-Ring C-C Dist C52      -C57      .       1.42 Ang.
PLAT606_ALERT_4_G Solvent Accessible VOID(S) in Structure ........          ! Info
PLAT860_ALERT_3_G Number of Least-Squares Restraints .............         90 Note
PLAT910_ALERT_3_G Missing # of FCF Reflection(s) Below Theta(Min).          3 Note
PLAT912_ALERT_4_G Missing # of FCF Reflections Above STh/L=  0.600        613 Note
PLAT933_ALERT_2_G Number of OMIT Records in Embedded .res File ...          7 Note
PLAT941_ALERT_3_G Average HKL Measurement Multiplicity ...........        3.1 Low
PLAT951_ALERT_5_G Calculated (ThMax) and CIF-Reported Kmax Differ           7 Units
PLAT978_ALERT_2_G Number C-C Bonds with Positive Residual Density.          3 Info
```

0 **ALERT level A** = Most likely a serious problem - resolve or explain
0 **ALERT level B** = A potentially serious problem, consider carefully
20 **ALERT level C** = Check. Ensure it is not caused by an omission or oversight
12 **ALERT level G** = General information/check it is not something unexpected

0 ALERT type 1 CIF construction/syntax error, inconsistent or missing data
18 ALERT type 2 Indicator that the structure model may be wrong or deficient
5 ALERT type 3 Indicator that the structure quality may be low
8 ALERT type 4 Improvement, methodology, query or suggestion
1 ALERT type 5 Informative message, check

It is advisable to attempt to resolve as many as possible of the alerts in all categories. Often the minor alerts point to easily fixed oversights, errors and omissions in your CIF or refinement strategy, so attention to these fine details can be worthwhile. In order to resolve some of the more serious problems it may be necessary to carry out additional measurements or structure refinements. However, the purpose of your study may justify the reported deviations and the more serious of these should normally be commented upon in the discussion or experimental section of a paper or in the "special_details" fields of the CIF. checkCIF was carefully designed to identify outliers and unusual parameters, but every test has its limitations and alerts that are not important in a particular case may appear. Conversely, the absence of alerts does not guarantee there are no aspects of the results needing attention. It is up to the individual to critically assess their own results and, if necessary, seek expert advice.

Publication of your CIF in IUCr journals

A basic structural check has been run on your CIF. These basic checks will be run on all CIFs submitted for publication in IUCr journals (*Acta Crystallographica*, *Journal of Applied Crystallography*, *Journal of Synchrotron Radiation*); however, if you intend to submit to *Acta Crystallographica Section C* or *E* or *IUCrData*, you should make sure that full publication checks are run on the final version of your CIF prior to submission.

Publication of your CIF in other journals

Please refer to the *Notes for Authors* of the relevant journal for any special instructions relating to CIF submission.

PLATON version of 03/06/2021; check.def file version of 02/06/2021

Datablock 20200905b - ellipsoid plot

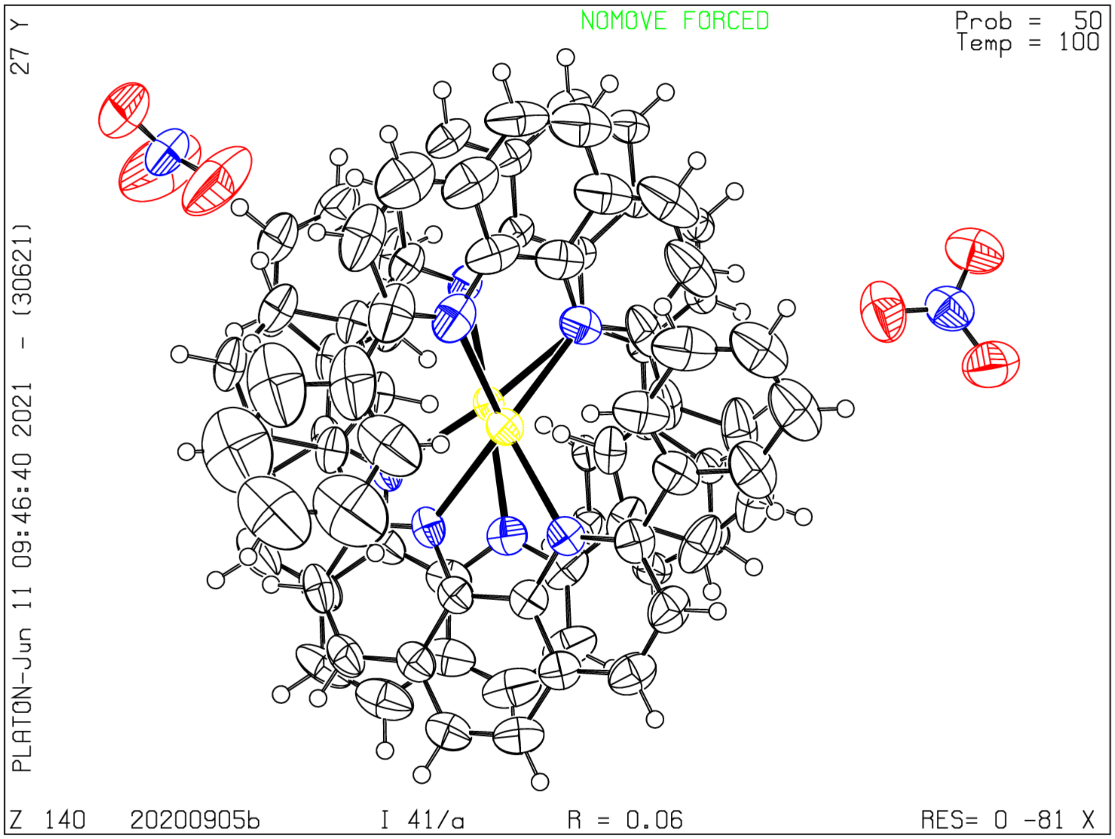

Supplement: Supplementary file 4 — Supplementary Data 1 [file 41467_2022_28921_MOESM4_ESM.zip › Crystallographic data/Checkcif.zip!/Checkcif/checkcif-Ag2-mdPPhen2.pdf]
