# Supplementary material for: In situ-formed tetrahedrally coordinated double-helical metal complexes for improved coordination-activated n-doping
Source: Nat Commun. 2022 Mar 8;13:1215. doi: 10.1038/s41467-022-28921-5 (PMC8904628; doi:10.1038/s41467-022-28921-5)

checkCIF/PLATON report

Structure factors have been supplied for datablock(s) bww18518

THIS REPORT IS FOR GUIDANCE ONLY. IF USED AS PART OF A REVIEW PROCEDURE FOR PUBLICATION, IT SHOULD NOT REPLACE THE EXPERTISE OF AN EXPERIENCED CRYSTALLOGRAPHIC REFEREE.

No syntax errors found. CIF dictionary Interpreting this report

Datablock: bww18518

Bond precision: C-C = 0.0038 A Wavelength=0.71073

Cell:	a=31.674(6)	b=12.338(3)	c=21.599(4)
	alpha=90	beta=119.14(3)	gamma=90
Temperature:	153 K		

	Calculated	Reported
Volume	7372(3)	7373(3)
Space group	C 2/c	C 1 2/c 1
Hall group	-C 2yc	-C 2yc
Moiety formula	C42 H26 N4, 2(C H2 Cl2), H2 O	2(C H2 Cl2), H2 O, C42 H26 N4
Sum formula	C44 H32 Cl4 N4 O	C44 H32 Cl4 N4 O
Mr	774.54	774.53
Dx,g cm-3	1.396	1.396
Z	8	8
Mu (mm-1)	0.363	0.363
F000	3200.0	3200.0
F000’	3205.74	
h,k,lmax	41,16,28	41,16,28
Nref	8442	8363
Tmin,Tmax	0.917,0.930	0.822,1.000
Tmin’	0.917	

Correction method= # Reported T Limits: Tmin=0.822 Tmax=1.000
AbsCorr = MULTI-SCAN

Data completeness= 0.991 Theta(max)= 27.486

R(reflections)= 0.0703(7507) wR2(reflections)= 0.1518(8363)

S = 1.164 Npar= 506

The following ALERTS were generated. Each ALERT has the format
test-name_ALERT_alert-type_alert-level.
Click on the hyperlinks for more details of the test.

Alert level C

```
PLAT336_ALERT_2_C Long Bond Distance for ..... C44A     -Cl3A          1.910 Ang.
PLAT906_ALERT_3_C Large K Value in the Analysis of Variance ......    13.505 Check
PLAT906_ALERT_3_C Large K Value in the Analysis of Variance ......     2.514 Check
PLAT910_ALERT_3_C Missing # of FCF Reflection(s) Below Theta(Min).         7 Note
PLAT911_ALERT_3_C Missing FCF Refl Between Thmin & STh/L=    0.600        46 Report
```

Alert level G

```
PLAT003_ALERT_2_G Number of Uiso or Uij Restrained non-H Atoms ...         6 Report
PLAT007_ALERT_5_G Number of Unrefined Donor-H Atoms .............          2 Report
PLAT012_ALERT_1_G No       _shelx_res_checksum Found in CIF ......    Please Check
PLAT042_ALERT_1_G Calc. and Reported Moiety Formula Strings Differ    Please Check
PLAT083_ALERT_2_G SHELXL Second Parameter in WGHT  Unusually Large     11.64 Why ?
PLAT128_ALERT_4_G Alternate Setting for Input Space Group     C2/c      I2/a Note
PLAT178_ALERT_4_G The CIF-Embedded .res File Contains SIMU Records         1 Report
PLAT186_ALERT_4_G The CIF-Embedded .res File Contains ISOR Records         1 Report
PLAT302_ALERT_4_G Anion/Solvent/Minor-Residue Disorder (Resd  3  )      100% Note
PLAT302_ALERT_4_G Anion/Solvent/Minor-Residue Disorder (Resd  4  )      100% Note
PLAT304_ALERT_4_G Non-Integer Number of Atoms in ..... (Resd  3  )      4.14 Check
PLAT304_ALERT_4_G Non-Integer Number of Atoms in ..... (Resd  4  )      0.86 Check
PLAT333_ALERT_2_G Large Aver C6-Ring C-C Dist C28      -C33      .      1.42 Ang.
PLAT411_ALERT_2_G Short Inter H...H Contact  H42      ..H44D     .      2.05 Ang.
                                         1/2-x,-1/2+y,1/2-z  =      4_545 Check
PLAT432_ALERT_2_G Short Inter X...Y Contact  N3       ..C44A            3.05 Ang.
                                         1/2-x,-1/2+y,1/2-z  =      4_545 Check
PLAT860_ALERT_3_G Number of Least-Squares Restraints ............         72 Note
PLAT912_ALERT_4_G Missing # of FCF Reflections Above STh/L=  0.600        27 Note
PLAT913_ALERT_3_G Missing # of Very Strong Reflections in FCF ....         2 Note
PLAT933_ALERT_2_G Number of OMIT Records in Embedded .res File ...        10 Note
PLAT941_ALERT_3_G Average HKL Measurement Multiplicity ..........        2.9 Low
PLAT978_ALERT_2_G Number C-C Bonds with Positive Residual Density.        14 Info
```

---

0 **ALERT level A** = Most likely a serious problem - resolve or explain
0 **ALERT level B** = A potentially serious problem, consider carefully
5 **ALERT level C** = Check. Ensure it is not caused by an omission or oversight
21 **ALERT level G** = General information/check it is not something unexpected

2 ALERT type 1 CIF construction/syntax error, inconsistent or missing data
8 ALERT type 2 Indicator that the structure model may be wrong or deficient
7 ALERT type 3 Indicator that the structure quality may be low
8 ALERT type 4 Improvement, methodology, query or suggestion
1 ALERT type 5 Informative message, check

It is advisable to attempt to resolve as many as possible of the alerts in all categories. Often the minor alerts point to easily fixed oversights, errors and omissions in your CIF or refinement strategy, so attention to these fine details can be worthwhile. In order to resolve some of the more serious problems it may be necessary to carry out additional measurements or structure refinements. However, the purpose of your study may justify the reported deviations and the more serious of these should normally be commented upon in the discussion or experimental section of a paper or in the "special_details" fields of the CIF. checkCIF was carefully designed to identify outliers and unusual parameters, but every test has its limitations and alerts that are not important in a particular case may appear. Conversely, the absence of alerts does not guarantee there are no aspects of the results needing attention. It is up to the individual to critically assess their own results and, if necessary, seek expert advice.

Publication of your CIF in IUCr journals

A basic structural check has been run on your CIF. These basic checks will be run on all CIFs submitted for publication in IUCr journals (*Acta Crystallographica*, *Journal of Applied Crystallography*, *Journal of Synchrotron Radiation*); however, if you intend to submit to *Acta Crystallographica Section C* or *E* or *IUCrData*, you should make sure that full publication checks are run on the final version of your CIF prior to submission.

Publication of your CIF in other journals

Please refer to the *Notes for Authors* of the relevant journal for any special instructions relating to CIF submission.

PLATON version of 03/06/2021; check.def file version of 02/06/2021

Datablock bww18518 - ellipsoid plot

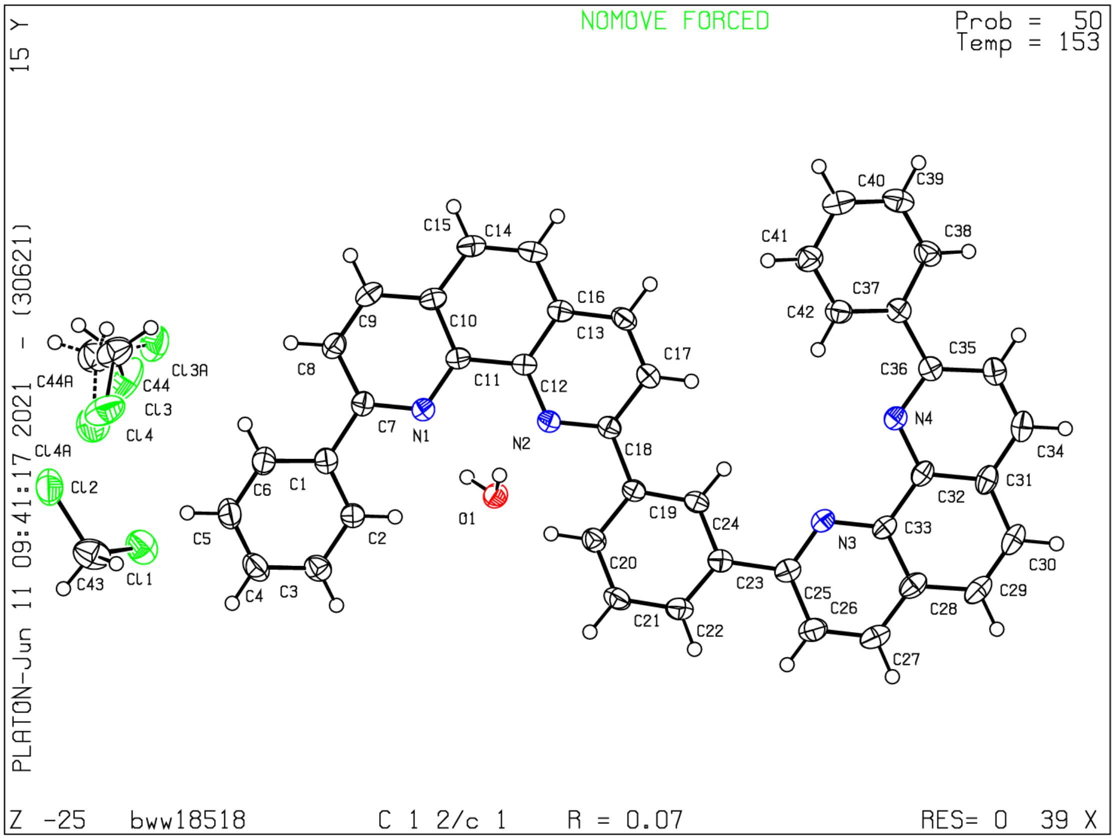

Supplement: Supplementary file 4 — Supplementary Data 1 [file 41467_2022_28921_MOESM4_ESM.zip › Crystallographic data/Checkcif.zip!/Checkcif/checkcif-m-dPPhen.pdf]
