# Supplementary material for: In situ-formed tetrahedrally coordinated double-helical metal complexes for improved coordination-activated n-doping
Source: Nat Commun. 2022 Mar 8;13:1215. doi: 10.1038/s41467-022-28921-5 (PMC8904628; doi:10.1038/s41467-022-28921-5)

checkCIF/PLATON report

Structure factors have been supplied for datablock(s) a

THIS REPORT IS FOR GUIDANCE ONLY. IF USED AS PART OF A REVIEW PROCEDURE FOR PUBLICATION, IT SHOULD NOT REPLACE THE EXPERTISE OF AN EXPERIENCED CRYSTALLOGRAPHIC REFEREE.

No syntax errors found. CIF dictionary Interpreting this report

Datablock: a

```
Bond precision:  C-C = 0.0054 A          Wavelength=0.71073

Cell:             a=16.915(3)     b=9.4251(19)     c=31.043(6)
                  alpha=90        beta=90          gamma=90
Temperature:      163 K

                Calculated                 Reported
Volume          4949.1(16)                 4948.9(17)
Space group     P b c a                    P b c a
Hall group      -P 2ac 2ab                 -P 2ac 2ab
Moiety formula  C30 H18 N4, C H2 Cl2       C H2 Cl2, C30 H18 N4
Sum formula     C31 H20 Cl2 N4             C31 H20 Cl2 N4
Mr              519.41                     519.41
Dx,g cm-3       1.394                      1.394
Z               8                          8
Mu (mm-1)       0.292                      0.292
F000            2144.0                     2144.0
F000'           2147.02
h,k,lmax        20,11,36                   20,11,36
Nref            4353                       4335
Tmin,Tmax       0.972,0.986                0.704,1.000
Tmin'           0.949

Correction method= # Reported T Limits: Tmin=0.704 Tmax=1.000
AbsCorr = MULTI-SCAN

Data completeness= 0.996          Theta(max)= 25.000

R(reflections)= 0.0760( 3670)     wR2(reflections)= 0.1507( 4335)

S = 1.212                    Npar= 334
```

The following ALERTS were generated. Each ALERT has the format
test-name_ALERT_alert-type_alert-level.
Click on the hyperlinks for more details of the test.

Alert level C

```
PLAT244_ALERT_4_C Low    'Solvent' Ueq as Compared to Neighbors of        C16 Check
PLAT340_ALERT_3_C Low Bond Precision on  C-C Bonds ...............    0.00541 Ang.
PLAT906_ALERT_3_C Large K Value in the Analysis of Variance ......     20.689 Check
PLAT906_ALERT_3_C Large K Value in the Analysis of Variance ......      3.226 Check
PLAT911_ALERT_3_C Missing FCF Refl Between Thmin & STh/L=    0.595         18 Report
```

Alert level G

```
PLAT003_ALERT_2_G Number of Uiso or Uij Restrained non-H Atoms ...         37 Report
PLAT042_ALERT_1_G Calc. and Reported Moiety Formula Strings Differ     Please Check
PLAT178_ALERT_4_G The CIF-Embedded .res File Contains SIMU Records          1 Report
PLAT186_ALERT_4_G The CIF-Embedded .res File Contains ISOR Records          1 Report
PLAT333_ALERT_2_G Large Aver C6-Ring C-C Dist C11      -C24      .       1.42 Ang.
PLAT860_ALERT_3_G Number of Least-Squares Restraints .............        474 Note
PLAT909_ALERT_3_G Percentage of I>2sig(I) Data at Theta(Max) Still        72% Note
PLAT933_ALERT_2_G Number of OMIT Records in Embedded .res File ...          4 Note
PLAT941_ALERT_3_G Average HKL Measurement Multiplicity ...........        3.7 Low
PLAT978_ALERT_2_G Number C-C Bonds with Positive Residual Density.          4 Info
```

---

0 **ALERT level A** = Most likely a serious problem - resolve or explain
0 **ALERT level B** = A potentially serious problem, consider carefully
5 **ALERT level C** = Check. Ensure it is not caused by an omission or oversight
10 **ALERT level G** = General information/check it is not something unexpected

1 ALERT type 1 CIF construction/syntax error, inconsistent or missing data
4 ALERT type 2 Indicator that the structure model may be wrong or deficient
7 ALERT type 3 Indicator that the structure quality may be low
3 ALERT type 4 Improvement, methodology, query or suggestion
0 ALERT type 5 Informative message, check

It is advisable to attempt to resolve as many as possible of the alerts in all categories. Often the minor alerts point to easily fixed oversights, errors and omissions in your CIF or refinement strategy, so attention to these fine details can be worthwhile. In order to resolve some of the more serious problems it may be necessary to carry out additional measurements or structure refinements. However, the purpose of your study may justify the reported deviations and the more serious of these should normally be commented upon in the discussion or experimental section of a paper or in the "special_details" fields of the CIF. checkCIF was carefully designed to identify outliers and unusual parameters, but every test has its limitations and alerts that are not important in a particular case may appear. Conversely, the absence of alerts does not guarantee there are no aspects of the results needing attention. It is up to the individual to critically assess their own results and, if necessary, seek expert advice.

Publication of your CIF in IUCr journals

A basic structural check has been run on your CIF. These basic checks will be run on all CIFs submitted for publication in IUCr journals (*Acta Crystallographica*, *Journal of Applied Crystallography*, *Journal of Synchrotron Radiation*); however, if you intend to submit to *Acta Crystallographica Section C* or *E* or *IUCrData*, you should make sure that full publication checks are run on the final version of your CIF prior to submission.

Publication of your CIF in other journals

Please refer to the *Notes for Authors* of the relevant journal for any special instructions relating to CIF submission.

PLATON version of 03/06/2021; check.def file version of 02/06/2021

Datablock a - ellipsoid plot

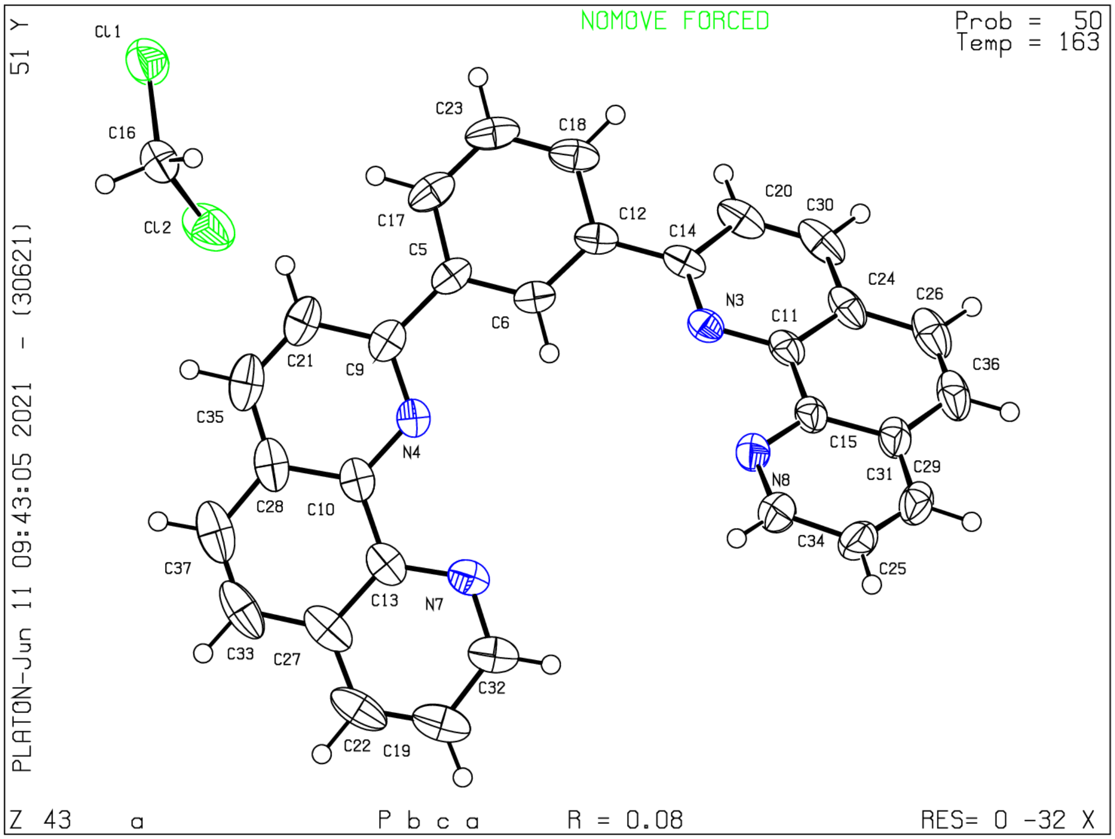

Supplement: Supplementary file 4 — Supplementary Data 1 [file 41467_2022_28921_MOESM4_ESM.zip › Crystallographic data/Checkcif.zip!/Checkcif/checkcif-m-dPhen-solvent evaporation.pdf]
