# Supplementary material for: In situ-formed tetrahedrally coordinated double-helical metal complexes for improved coordination-activated n-doping
Source: Nat Commun. 2022 Mar 8;13:1215. doi: 10.1038/s41467-022-28921-5 (PMC8904628; doi:10.1038/s41467-022-28921-5)

checkCIF/PLATON report

Structure factors have been supplied for datablock(s) a

THIS REPORT IS FOR GUIDANCE ONLY. IF USED AS PART OF A REVIEW PROCEDURE FOR PUBLICATION, IT SHOULD NOT REPLACE THE EXPERTISE OF AN EXPERIENCED CRYSTALLOGRAPHIC REFEREE.

No syntax errors found. CIF dictionary Interpreting this report

Datablock: a

```
Bond precision:  C-C = 0.0042 A          Wavelength=0.71073

Cell:              a=35.472(5)      b=35.472(5)      c=12.264(3)
                   alpha=90         beta=90          gamma=120
Temperature:       165 K

                Calculated                 Reported
Volume          13364(5)                   13363(5)
Space group     R -3                       R -3
Hall group      -R 3                       -R 3
Moiety formula  C42 H26 N4                 C42 H26 N4
Sum formula     C42 H26 N4                 C42 H26 N4
Mr              586.67                     586.67
Dx,g cm-3       1.312                      1.312
Z               18                         18
Mu (mm-1)       0.078                      0.078
F000            5508.0                     5508.0
F000'           5509.92
h,k,lmax        42,42,14                   42,42,14
Nref            5236                       5213
Tmin,Tmax       0.986,0.989                0.865,1.000
Tmin'           0.986

Correction method= # Reported T Limits: Tmin=0.865 Tmax=1.000
AbsCorr = MULTI-SCAN

Data completeness= 0.996          Theta(max)= 24.998

R(reflections)= 0.0818( 4937)     wR2(reflections)= 0.1567( 5213)

S = 1.364                  Npar= 415
```

The following ALERTS were generated. Each ALERT has the format
test-name_ALERT_alert-type_alert-level.
Click on the hyperlinks for more details of the test.

Alert level C

```
PLAT340_ALERT_3_C Low Bond Precision on  C-C Bonds ...............    0.00415 Ang.
PLAT906_ALERT_3_C Large K Value in the Analysis of Variance ......     22.665 Check
PLAT906_ALERT_3_C Large K Value in the Analysis of Variance ......      3.462 Check
PLAT911_ALERT_3_C Missing FCF Refl Between Thmin & STh/L=    0.595         20 Report
```

Alert level G

```
PLAT083_ALERT_2_G SHELXL Second Parameter in WGHT  Unusually Large      30.24 Why ?
PLAT909_ALERT_3_G Percentage of I>2sig(I) Data at Theta(Max) Still        93% Note
PLAT910_ALERT_3_G Missing # of FCF Reflection(s) Below Theta(Min).          3 Note
PLAT933_ALERT_2_G Number of OMIT Records in Embedded .res File ...          5 Note
PLAT978_ALERT_2_G Number C-C Bonds with Positive Residual Density.          5 Info
```

0 **ALERT level A** = Most likely a serious problem - resolve or explain
0 **ALERT level B** = A potentially serious problem, consider carefully
4 **ALERT level C** = Check. Ensure it is not caused by an omission or oversight
5 **ALERT level G** = General information/check it is not something unexpected

0 ALERT type 1 CIF construction/syntax error, inconsistent or missing data
3 ALERT type 2 Indicator that the structure model may be wrong or deficient
6 ALERT type 3 Indicator that the structure quality may be low
0 ALERT type 4 Improvement, methodology, query or suggestion
0 ALERT type 5 Informative message, check

It is advisable to attempt to resolve as many as possible of the alerts in all categories. Often the minor alerts point to easily fixed oversights, errors and omissions in your CIF or refinement strategy, so attention to these fine details can be worthwhile. In order to resolve some of the more serious problems it may be necessary to carry out additional measurements or structure refinements. However, the purpose of your study may justify the reported deviations and the more serious of these should normally be commented upon in the discussion or experimental section of a paper or in the "special_details" fields of the CIF. checkCIF was carefully designed to identify outliers and unusual parameters, but every test has its limitations and alerts that are not important in a particular case may appear. Conversely, the absence of alerts does not guarantee there are no aspects of the results needing attention. It is up to the individual to critically assess their own results and, if necessary, seek expert advice.

Publication of your CIF in IUCr journals

A basic structural check has been run on your CIF. These basic checks will be run on all CIFs submitted for publication in IUCr journals (*Acta Crystallographica*, *Journal of Applied Crystallography*, *Journal of Synchrotron Radiation*); however, if you intend to submit to *Acta Crystallographica Section C* or *E* or *IUCrData*, you should make sure that full publication checks are run on the final version of your CIF prior to submission.

Publication of your CIF in other journals

Please refer to the *Notes for Authors* of the relevant journal for any special instructions relating to CIF submission.

PLATON version of 03/06/2021; check.def file version of 02/06/2021

Datablock a - ellipsoid plot

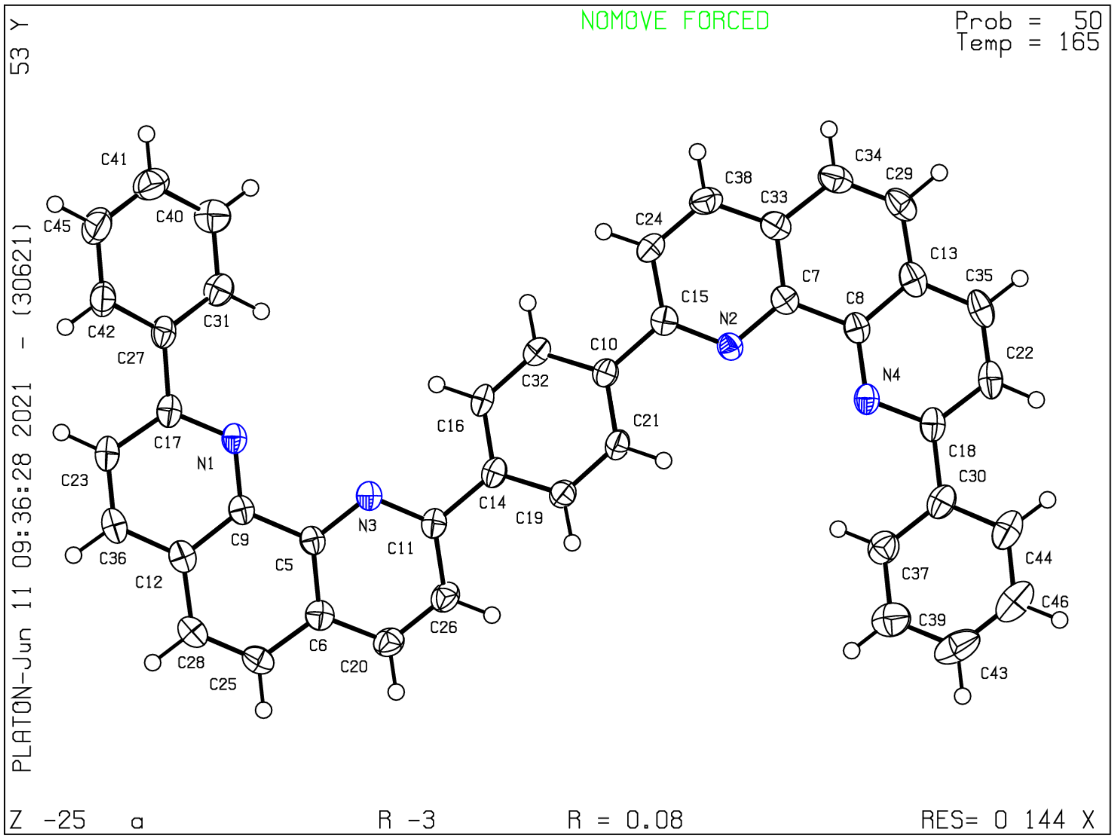

Supplement: Supplementary file 4 — Supplementary Data 1 [file 41467_2022_28921_MOESM4_ESM.zip › Crystallographic data/Checkcif.zip!/Checkcif/checkcif-p-dPPhen.pdf]
